# Anode‐Free All‐Solid‐State Batteries: Understanding Limitations and Charting a Path to Enhanced Performance

**DOI:** 10.1002/smll.74019

**Published:** 2026-06-02

**Authors:** Sion Kim, Jaechan Lee, Jihyun Jang

**Affiliations:** ^1^ Department of Chemistry Sogang University Seoul Republic of Korea; ^2^ Center for Nano Materials Sogang University Seoul Republic of Korea

**Keywords:** all‐solid‐state batteries, anode‐free, interlayer engineering, cell assembly and conditions, pressure‐regulating buffer layers, sacrificial cathodes, solid‐state electrolyte modification, sulfide‐based solid‐state electrolyte

## Abstract

All‐solid‐state batteries (ASSBs) utilizing sulfide solid‐state electrolytes (SSEs) emerge as the most promising platform for next‐generation, high‐energy‐density systems, owing to the exceptional ionic conductivity of SSEs. To maximize energy density, anode‐free ASSBs involving direct Li plating and stripping onto the current collector are actively explored. However, the absence of an excess lithium source exacerbates interfacial instability between the SSE and the current collector, impeding commercial viability. This review analyzes the chemical, thermal, electrochemical, and mechanical vulnerabilities of Anode‐free sulfide ASSBs. Chemically, atmospheric exposure generates toxic H_2_S gas and corrodes the current collector. Furthermore, the narrow electrochemical stability window necessitates the formation of an SEI‐like layer composed of decomposition products. Non‐uniform electronic and ionic conductivity within this SEI causes localized current density, ultimately promoting Li dendrite growth. Mechanically, non‐uniform Li plating‐stripping dynamics under high current density accelerate the accumulation of dead Li, while inadequate stack pressure and the significant volume changes during cycling deteriorate cycling stability. To overcome these multifaceted challenges, this study emphasizes the need to develop sophisticated interfacial engineering strategies, alongside active pressure management systems that accommodate volume variations.

## Graphical abstract

This review examines four factors that drive interfacial instability in sulfide‐based anode‐free all‐solid‐state batteries and thoroughly reviews corresponding performance‐enhancing strategies. The strategies are organized as follows: (1) interlayer engineering, (2) pressure‐regulating buffer layers, (3) other methods, including sacrificial cathodes, modification of solid‐state electrolytes, and cell assembly and cycle conditions.

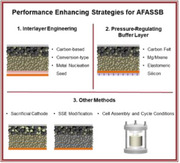

## 1 Introduction

The rapid expansion of electric vehicles (EVs) and grid‐scale energy storage systems (ESSs) necessitates rechargeable batteries that simultaneously deliver higher energy density and uncompromised safety. Conventional Lithium‐ion batteries (LIBs) rely on organic liquid electrolytes that are highly reactive toward Li metal; consequently, graphite (372 mAh g^−1^) or silicon‐graphite composite anodes are typically employed, imposing intrinsic limits on achieving higher energy density [1, 2, 3]. In addition, organic liquid electrolytes can act as combustible fuels, raising fundamental safety constraints [4, 5].

All‐solid‐state batteries (ASSBs) have emerged as a promising alternative by replacing flammable liquids with nonflammable solid‐state electrolytes (SSEs) [6]. Representative SSE families include oxides (e.g., garnet Li_7_La_3_Zr_2_O_12_ (LLZO), perovskite Li_3.3_La_0.56_TiO_3_, NASICON‐type Li_1.5_Al_0.5_Ge_1.5_(PO_4_)_3_, and LISICON‐type Li_3.5_Si_0.5_P_0.5_O_4_), sulfides (e.g., Li_2_S‐P_2_S_5_ and Li_10_GeP_2_S_12_, Li_6_PS_5_Cl (LPSCl), and organic polymeric systems consisting of a polymer matrix (e.g., poly(ethylene oxide), poly(vinylidene fluoride), and polyacrylonitrile) and Li salts (e.g., LiClO_4_, and LiSO_3_CF_3_) [7, 8, 9, 10, 11, 12]. In addition to its nonflammability, SSEs feature a wide electrochemical stability window and high ionic conductivity at room temperature [13]. Beyond safety features, ASSBs enable Li metal, featuring the lowest electrochemical potential of −3.04 V (vs. standard hydrogen electrode) and an ultrahigh theoretical specific capacity of 3860 mAh g^−1^, offering a viable path toward substantially higher energy density [14].

More recently, anode‐free all‐solid‐state batteries (AFASSBs), also referred to as “anode‐less”, “Li‐free”, or “reservoir free”, have attracted growing interest across academia and industry. In AFASSB architectures, no excess Li is incorporated during cell fabrication; metallic Li is formed in situ on the bare current collector during the initial charging step, supplied solely by the cathode [15, 16]. The subsequent cycling after the initial charge–discharge proceeds in the same manner as that of conventional ASSBs. Owing to this distinct cell configuration, several different terms have been used interchangeably in the literature. For clarity and consistency, this review adopts the term “anode‐free” throughout. Eliminating pre‐deposited Li increases both gravimetric and volumetric energy densities, and ASSBs incorporating thin SSEs, rather than a separator membrane used in anode‐free lithium metal batteries (AFLMBs), can further enhance energy density. Beyond enhancing energy density, AFASSBs inherit the intrinsic safety of solid‐state systems, cost reduction from omitting Li metal, and simplified fabrication by eliminating Li handling. AFASSBs are not only beneficial for safety and cost‐effectiveness but also simplify the manufacturing process, thereby enhancing scalability [17].

**FIGURE 1:**
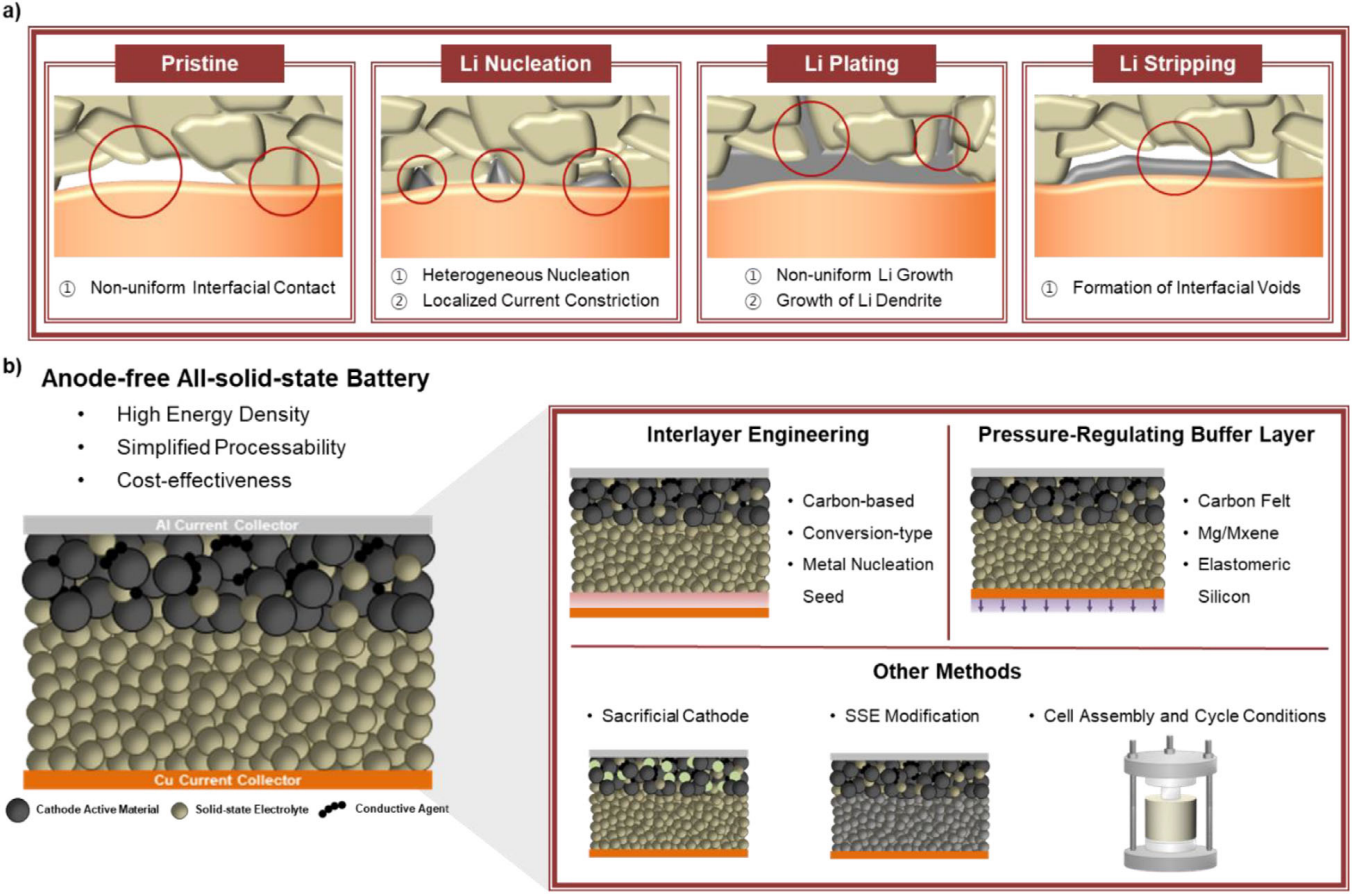
(a) Cascading degradation mechanisms in Anode‐free All‐solid‐state Batteries (AFASSBs), (b) Schematic diagrams of AFASSBs and various performance‐enhancing strategies for AFASSBs.

Despite these advantages, AFASSBs face nontrivial challenges. Nonuniform Li^+^ flux and current constriction during plating promote dendritic growth and the accumulation of electrically isolated “dead Li” [18, 19]. During stripping, contact loss at the SSE‖current collector interface and void formation increase impedance and accelerate capacity fade (Figure 1a) [20]. In sulfide SSEs, our focus in this review, chemo‐mechanical fragility under volume changes can lead to microcracking [21], intergranular Li penetration, and inadvertent electronic percolation pathways [22], thereby increasing the risk of short circuits. Moreover, because AFASSBs operate with a limited Li inventory, any irreversible loss directly accelerates energy depletion [23].

This review critically examines sulfide‐based AFASSBs, organized into two major sections: (1) performance‐limiting factors, and (2) key strategies to enhance performance in AFASSBs (Figure 1b). By providing an in‐depth analysis of the failure mechanisms in AFASSBs and corresponding mitigation strategies, this review intends to serve as a guideline for researchers to design high‐performance AFASSBs more effectively.

## 2 Performance‐Limiting Factors in Anode‐Free All‐Solid‐State Batteries

Sulfide‐based SSEs have emerged as up‐and‐coming candidates for ASSBs because of their high ionic conductivity, favorable mechanical softness, and excellent processability [24]. Sulfide‐based electrolytes are considered the closest to practical commercialization among various solid electrolyte systems. However, despite remarkable progress in their electrochemical performance, large‐scale implementation remains challenging. This is particularly critical in AFASSB configurations, where the interfacial stability between the current collector and the SSE directly determines the reversibility of lithium plating/stripping and long‐term cycling stability. The interfacial stability of sulfide SSEs is governed by multiple factors, including chemical, thermal, electrochemical, and mechanical stability during cell assembly and operation. Although this review primarily focuses on sulfide‐based systems, selected studies on other solid electrolytes, including garnet‐type LLZO, NASICON‐type, LISICON‐type, and LiPON (Li_x_PO_y_N_z_), are also discussed to broaden understanding of anode‐free ASSBs and to provide additional insight into interfacial phenomena and failure mechanisms shared across solid‐state battery systems.

### 2.1 Interfacial Instability of Sulfide‐Based Solid‐State Electrolytes

#### 2.1.1 Chemical Stability

The chemical stability of sulfide SSEs under ambient conditions is a significant issue that directly affects their processability and interfacial characteristics. Since the first report on the air stability of sulfide electrolytes by M. Tatsumisago in 2011, extensive studies have shown that sulfide electrolytes are highly sensitive to moisture and oxygen [25, 26]. In particular, electrolytes containing P_2_S_7_^4–^ structural units, such as Li_7_P_3_S_11_, exhibit faster hydrolysis than PS_4_^3–^‐based electrolytes, which show relatively superior air stability [27].

**FIGURE 2:**
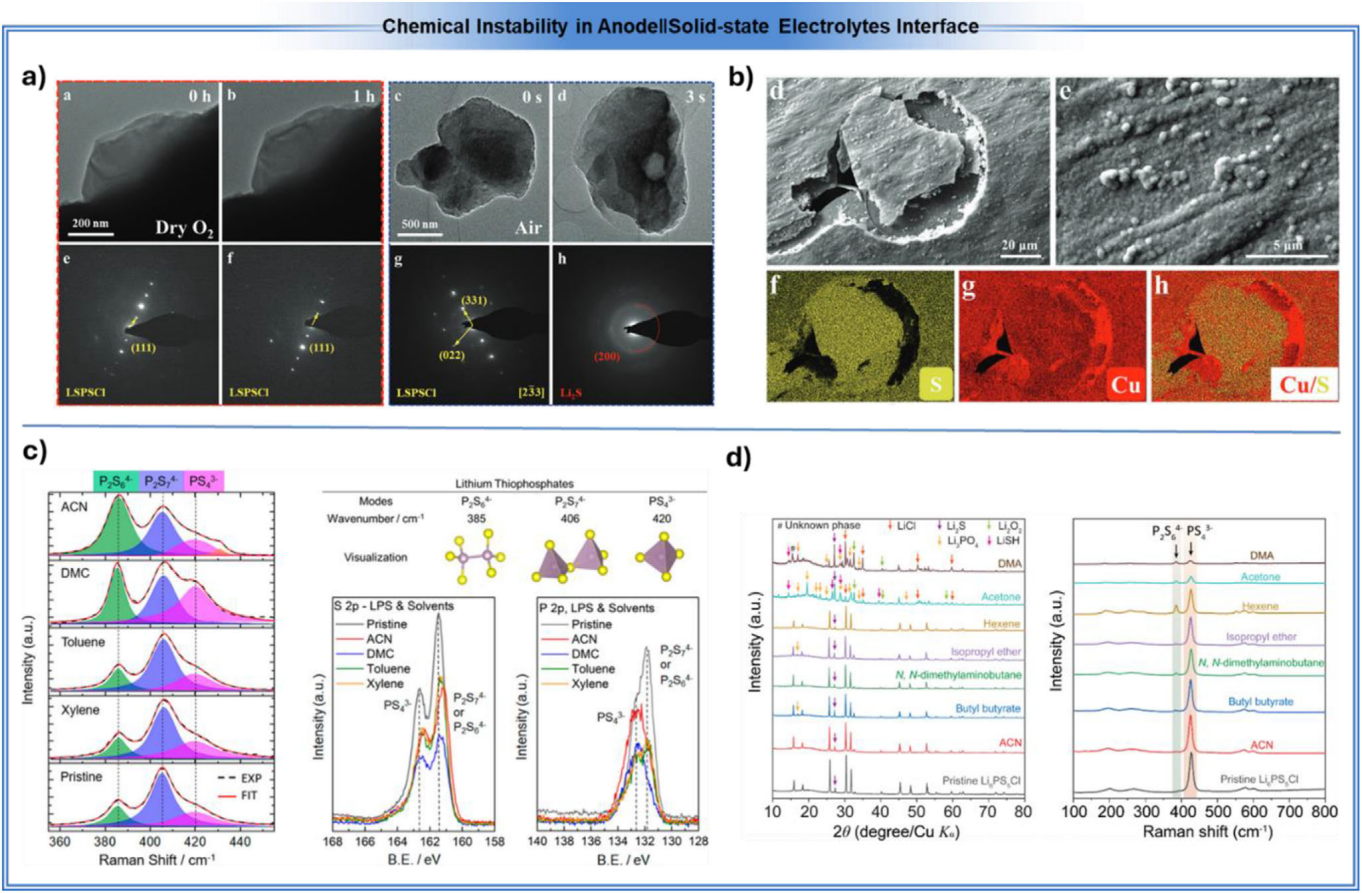
Interfacial instabilities of sulfide‐based SSEs. (a) In situ TEM observation of a sulfide SSE after atmospheric exposure, illustrating its chemical instability. (b) SEM image and corresponding EDX mapping of a Cu current collector surface. A small amount of moisture reacts with LPSCl, releasing H_2_S gas that induces Cu corrosion and subsequent Cu_2_S formation. Reproduced with permission [28]. Copyright 2024, Wiley‐VCH. (c) Raman spectra of Li_7_P_3_S_11_ after solvent dispersion and XPS spectra of Li thiophosphate. These results clearly demonstrate that solvent selection is critical when processing Li_7_P_3_S_11_ SEs via solvent‐based fabrication. Reproduced with permission [29]. Copyright 2019, American Chemical Society. (d) Stability of LPSCl SE immersed in various organic solvents, verified through XRD patterns and Raman spectra. Reproduced with permission [30]. Copyright 2024, Wiley‐VCH.

Moisture exposure leads to a marked deterioration of interfacial properties. In Figure 2a, in situ transmission electron microscopy (TEM) revealed a pronounced decrease in crystallinity and an increase in amorphous regions, even after short‐term air exposure, compared to samples stored in a dry room. The hydrolysis of sulfide SSEs generates H_2_S gas, which reacts with copper current collectors to form copper sulfides (e.g., CuS), as confirmed by scanning electron microscopy energy dispersive X‐ray spectroscopy (SEM‐EDS) mapping in Figure 2b. This corrosion is particularly problematic in anode‐free configurations, where the SSE directly contacts the Cu collector without a protective anode layer [28].

H_2_S also corrodes other metallic components and is highly cytotoxic, posing additional safety concerns for both battery modules and their surrounding infrastructure [31]. Even cells fabricated under dry‐room conditions with a dew point of −50°C have shown Cu surface degradation caused by trace moisture reacting with LPSCl to generate H_2_S [28]. These studies highlight the urgent need for improved ambient stability of sulfide electrolytes and strict environmental control during cell fabrication to suppress interfacial degradation.

For large‐scale fabrication of SSEs, slurry‐based processing has been proposed to produce thin‐film solid electrolyte membranes [32]. During the slurry preparation of solid electrolyte membranes or composite electrodes, the solvents and binders used can chemically interact with the solid electrolyte, potentially affecting its structural integrity and interfacial stability in anode‐free configurations. Solvents that chemically react with sulfide‐based solid electrolytes may alter their crystal structure and severely degrade ionic conductivity, making them unsuitable for slurry processing [27].

Figure 2c shows Raman spectra of Li_7_P_3_S_11_‐based sulfide electrolytes prepared by wet casting with various solvents [29]. Each mode and its wavenumber of pristine and decomposed sulfide are also described. Compared with the pristine Li_7_P_3_S_11_, samples processed with acetonitrile (ACN) and dimethyl carbonate (DMC) exhibited a decrease in the P_2_S_7_^4−^ peak (406 cm^−1^) and an increase in peaks corresponding to decomposition products such as P_2_S_6_^4–^ (385 cm^−1^) and PS_4_^3−^ (420 cm^−1^), indicating solvent‐induced degradation of the electrolyte. In contrast, samples processed with toluene or xylene showed no change in Raman features relative to the pristine sample, suggesting minimal chemical interaction with the sulfide phase. X‐ray photoelectron spectroscopy (XPS) further confirmed these results: in the P 2p region, samples dispersed in ACN and DMC showed a blue shift toward higher binding energies, consistent with the formation of PS_4_^3−^ species. These observations indicate that high‐dielectric‐constant solvents such as ACN and DMC promote the conversion of P_2_S_7_^4−^ to PS_4_^3−^, whereas low‐polarity, low‐dielectric‐constant solvents such as toluene and xylene exhibit superior chemical compatibility with sulfide electrolytes.

According to a study by Zhao et al., severe electrolyte decomposition occurred in acetone and N,N‐dimethylacetamide (DMA) when LPSCl was used as the SSE [30]. As shown in Figure 2d, both acetone and DMA induced pronounced structural degradation, evidenced by the emergence of multiple new peaks in the X‐ray diffraction (XRD) patterns compared with the pristine LPSCl. Raman spectra further confirmed these results, showing diminished PS_4_^3–^ peaks and the generation of P_2_S_6_^4–^ species, indicative of electrolyte decomposition. The authors attributed this degradation to the polar, oxygen‐ and nitrogen‐containing functional groups (carbonyl and amide) in acetone and DMA, whose electronegative oxygen and nitrogen atoms interact with P^5+^ in the LPSCl framework, leading to structural breakdown and the formation of poorly conductive products. In contrast to the results shown in Figure 2c, the influence of ACN on the solid electrolyte was found to be negligible in Figure 2d. This is consistent with previous findings using tetra‐Li_7_SiPS_8_ as the SSE, where ACN was identified as a compatible solvent due to its low donor number [33]. These observations suggest that the applicability and chemical compatibility of solvents for slurry processing depend strongly on the specific composition of the sulfide‐based SSEs.

#### 2.1.2 Thermal Stability

**FIGURE 3:**
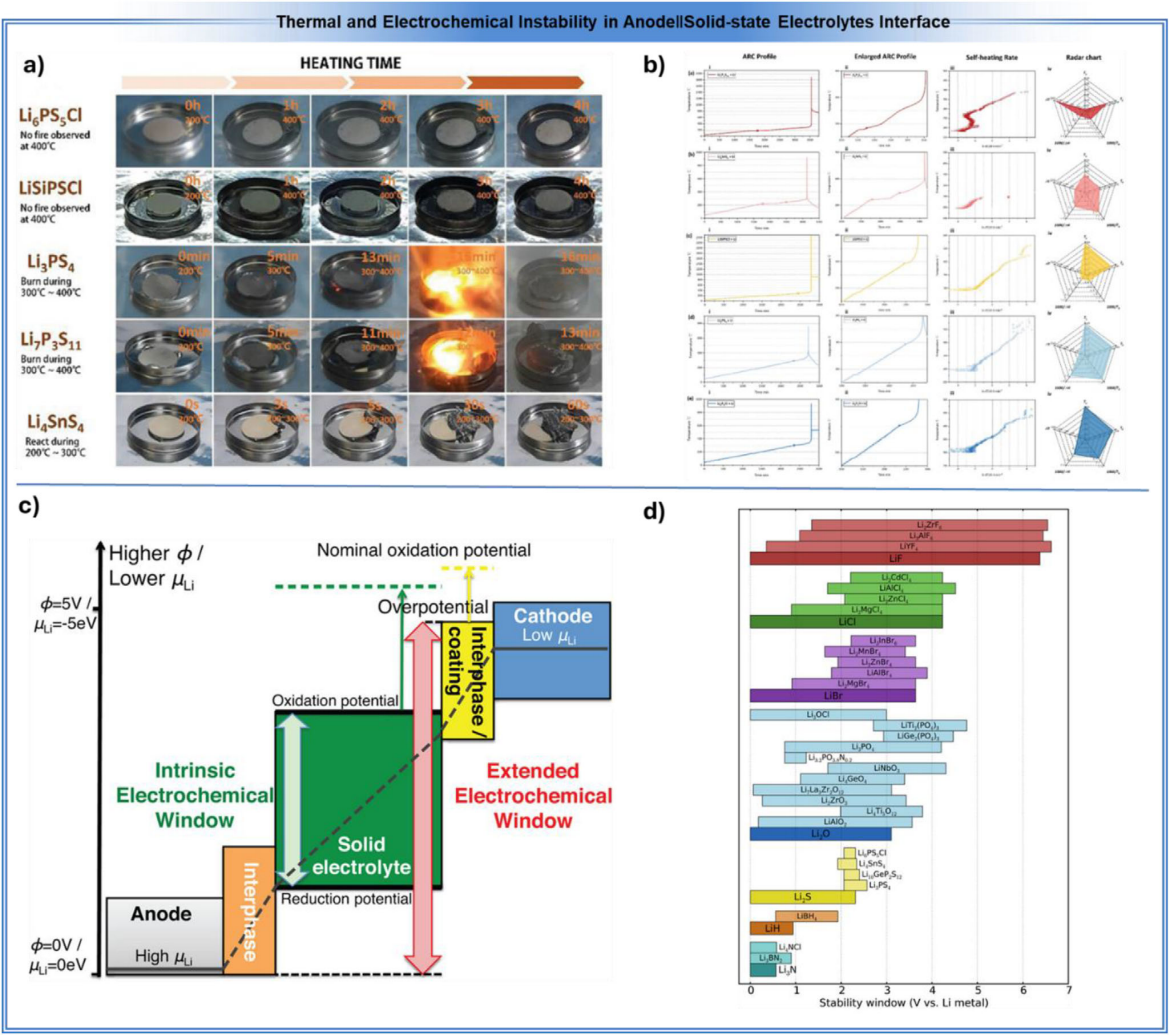
Thermal and Electrochemical Instability of SSE Interface. (a) Thermal instability of sulfide SSEs and Li metal. Although sulfide SSEs are less flammable than conventional liquid electrolytes, they exhibited flammability above 400°C. (b) ARC analysis of different sulfide SSEs and Li metal, showing heat suppression behavior for Li7P3S11 and thermal vulnerability for Li4SnS4. Reproduced with permission [34]. Copyright 2023, Wiley‐VCH. (c) Schematic illustration showing that the interfacial passivation layer effectively extends the ESW of SSEs, thereby preventing further decomposition. Reproduced with permission [35]. Copyright 2015, American Chemical Society. (d) Electrochemical stability windows of various SSEs and their binary‐anion counterparts. Sulfide‐based SSEs, characterized by a narrow stability window, react with Li+ to form stable passivating interphases that suppress further decomposition. Adapted with permission [36]. Copyright 2016, American Chemical Society.

Thermal stability, particularly at the interface between sulfide SSEs and the current collector, represents another crucial factor in anode‐free cell design. When the thermal stability of all battery components in sulfide‐based all‐solid‐state batteries is considered comprehensively, cathode active materials are often regarded as among the most thermally vulnerable components, and their decomposition temperature tends to decrease with increasing Ni content and high SOCs [37]. In addition, cells employing Li metal as the anode inherently suffer from limited thermal stability because Li melts at around 180°C, and this concern also applies to anode‐free systems, where Li is plated in situ during charging [38]. While solid electrolytes are less flammable than liquid electrolytes, their thermal decomposition behavior varies significantly with composition. Figure 3a compares the thermal behavior of several sulfide compositions. LPSCl and Li_9.54_Si_1.74_P_1.44_S_11.7_Cl_0.3_ (LiSiPSCl) showed exceptional stability, with no detectable changes even after heating at 400°C for 4 h. In contrast, Li_3_PS_4_ and Li_7_P_3_S_11_ ignited within 10 min under the same conditions, and similar exothermic behavior was observed during frictional heating in grinding experiments. Accelerating Rate Calorimeter (ARC) measurements were performed at the SSE‖Li interface to assess further interfacial thermal behavior. Figure 3b shows that Li_4_SnS_4_, although known as thermally stable, ignited earliest because of severe interfacial instability with Li metal. By contrast, Li_7_P_3_S_11_ exhibited delayed decomposition, attributed to the formation of a Li_2_S‐rich interphase that temporarily suppressed self‐heating. While reduced lithium inventory in anode‐free systems is expected to improve overall thermal stability, these results emphasize that both bulk electrolyte stability and the robustness of the interfacial passivating film must be carefully engineered [34].

Meanwhile, the organic SEI formed in conventional LIBs is known to undergo thermal decomposition due to its limited thermal stability, with initial SEI decomposition reported to begin at approximately 60°C and exothermic reactions generally occurring near 80°C, which can eventually trigger thermal runaway [39]. In contrast, major reductive decomposition products formed at the interface between sulfide SSEs and the anode, such as Li_3_P, Li_2_S, and LiCl, are inorganic species that can form a thermally more robust interphase than the organic SEI typically formed in conventional LIBs [40]. Accordingly, compared with the organic SEI typically formed in conventional LIBs, the inorganic interphase formed in sulfide‐based ASSBs may offer superior thermal stability, thereby reducing the risk of thermal runaway [41]. Nevertheless, overall cell‐level thermal safety is governed not only by the thermal stability of the interphase itself, but also by the presence of Li metal, cathode oxygen release, interfacial contact conditions, and cell design.

#### 2.1.3 Electrochemical Stability

Sulfide SSEs possess a narrower electrochemical stability window (ESW) than oxide electrolytes, rendering them more susceptible to decomposition. As illustrated in Figure 3c, this often leads to the formation of passivation layers, such as the solid–electrolyte interphase (SEI) and the cathode–electrolyte interphase (CEI). The ESW of the overall cell can be effectively expanded by the wider stability window of these interphases, which suppresses further electrolyte decomposition [35]. The fundamental requirements for an ideal SEI, which are high ionic conductivity, low electronic conductivity, and mechanical stability, apply equally to ASSBs [42].

Lithium is directly plated on the Cu current collector in anode‐free systems, making electrolyte stability near 0 V (vs. Li/Li^+^) crucial. Most sulfide SSEs decompose at this potential, producing Li_2_S and Li_3_P, which form a passivation interphase, as shown in Figure 3d [36]. Compared with oxide SSEs, sulfide SSEs generally form more reactive and continuously growing interphases at low potentials, resulting in clear differences in the composition, transport characteristics, and growth behavior of SEI/CEI layers, which can have profound implications for anode‐free systems [43]. In oxide SSEs such as garnet‐type LLZO, the surface is often initially covered with air‐derived Li_2_CO_3_ species, which increase lithiophobicity and interfacial resistance even before cell operation [44, 45]. Upon contact with Li metal, LLZO is often described as exhibiting only very limited interfacial reactivity or forming a very thin lithiated interphase, suggesting kinetically constrained decomposition that suppresses further interfacial growth [43]. By contrast, sulfide SSEs undergo more extensive reductive decomposition, forming chemically heterogeneous interphases, and some of their decomposition products can exhibit partially mixed ionic‐electronic conducting character, thereby enabling continued parasitic reactions and progressive interphase thickening rather than immediate passivation [40, 43]. While this interphase allows operation outside the intrinsic ESW, its transport and mechanical properties differ from those of pristine SSEs, strongly impacting performance. If the interphase exhibits poor ionic transport or high electronic conductivity, parasitic reactions and accelerated lithium consumption can occur [40, 43]. Nonuniform interphase growth leads to local current hotspots, promoting dendrite growth and uneven Li deposition, which in turn cause internal short circuits and rapid capacity fading [46].

### 2.2 Non‐Uniform Li Plating and Stripping

Lithium plating and stripping behaviors are decisive for the performance of anode‐free batteries. Unlike conventional anodes such as graphite or silicon that store lithium through intercalation or alloying, anode‐free systems rely entirely on lithium deposition and dissolution, minimizing inactive mass and maximizing energy density. However, this also means that nonuniform deposition, dead lithium formation, and Coulombic inefficiencies can severely degrade cell performance. Therefore, a comprehensive understanding of the mechanisms governing lithium plating and stripping, along with the associated factors influencing these processes, is essential for sulfide‐based AFASSB systems.

Although sulfide‐based SSEs exhibit low electrochemical stability, the reduction products formed within their limited ESW can function as SEI layers that stabilize the interface, as discussed previously [35]. However, the composition, structure, and elemental makeup of sulfide SSEs can markedly influence the chemical nature and functionality of these SEI layers, leading to distinct interfacial behaviors. Therefore, SEI properties can affect the uniformity of plating/stripping in AFASSBs. In addition, in AFASSBs without any current collector surface treatment, Li must nucleate and grow directly on a bare current collector rather than a pre‐formed Li metal surface [47]. Due to the intrinsically poor lithiophilicity of most current collector metals, the nucleation energy barrier remains high, leading to spatially heterogeneous nucleation and growth. The resulting non‐uniform Li^+^ flux and current constriction promote dendritic protrusions, “dead Li” accumulation, and localized fields and stresses at the interface between the current collector and SSE [15, 48, 49]. These features collectively aggravate interfacial instability and make uniform Li deposition and dissolution more difficult.

**FIGURE 4:**
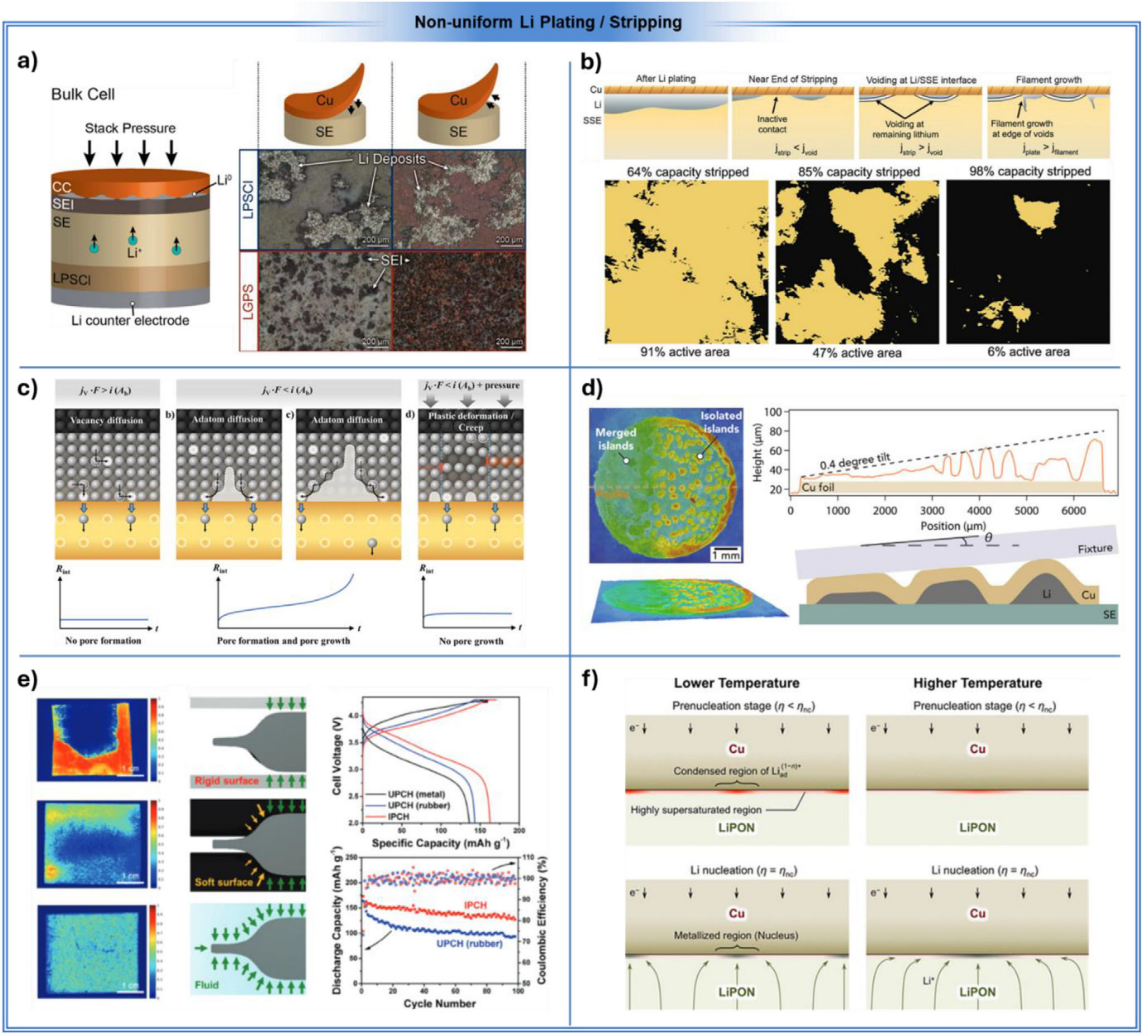
Interfacial and mechanical factors influencing uniform Li plating/stripping behavior in solid‐state cells. (a) Optical observation of interfacial characteristics in bulk Li/SSE/Cu cells, interfacial morphologies observed depending on the sulfide solid electrolyte type. Reproduced with permission [50]. Copyright 2021 The Electrochemical Society. (b) Void formation mechanism during lithium stripping and X‐ray tomography analysis of the active lithium area. Reproduced with permission [49]. Copyright 2023, Wiley‐VCH. (c) Stripping‐related interfacial degradation of Li metal anodes and the effect of stack pressure on interfacial contact. Reproduced with permission [51]. Copyright 2019, American Chemical Society. (d) Uneven Li plating on a Cu foil caused by tilted peak surfaces and non‐uniform pressure distribution, leading to morphological and thickness variations. Reproduced with permission [52]. Copyright 2022, Elsevier. (e) The application of interfacial pressure contact holders (IPCH) promotes uniform contact between the current collector and the solid electrolyte, enhancing overall cell performance. Reproduced with permission [53]. Copyright 2024, Wiley‐VCH. (f) Temperature‐induced variation in Li nucleation on current collectors. Adapted with permission [54]. Copyright 2020, American Chemical Society.

As shown in Figure 4a, N. P. Dasgupta group fabricated a bulk Li/LPSCl/SE/Cu cell and applied stack pressure during lithium plating, followed by post‐mortem optical analysis of the SSE‖Cu interface after the first plating process [50]. When LPSCl was used as the solid electrolyte, metallic silver‐colored lithium deposits were observed on both the SSE and Cu surfaces. In contrast, when L_i10_GeP_2_S_12_ (LGPS) was used as the SSE, dark spots of SEI decomposition products were distributed across the interface. Unlike the electron‐insulating decomposition products of LPSCl, the SEI compounds formed from LGPS possess higher electronic conductivity, which can trigger additional SSE decomposition and accelerate lithium consumption. These results indicate that the intrinsic electrochemical and chemical properties of sulfide‐based SSEs govern lithium plating behavior, underscoring the importance of electrolyte selection for stable interfacial performance in solid‐state cells.

Figure 4b illustrates a mechanism of performance degradation in AFASSBs from the perspective of lithium stripping, complementing the preceding discussion focused on uniform plating. The detachment between the lithium phosphorus oxynitride SSE and the current collector, as first noted by Neudecker et al. in the pioneering study, the concept of AFASSBs, also represents a critical factor hindering uniform lithium stripping [55]. When stripping occurs after uniform or non‐uniform lithium plating, uneven lithium dissolution leads to direct contact between the bare current collector and the SSE. At these inactive contact regions, further stripping cannot occur, leading to a concentration of local current density in lithium‐plated areas. This localized current density (j_strip_) eventually exceeds the critical threshold for void formation (j_void_), resulting in interfacial voids between the SSE and the remaining Li.

Upon subsequent plating, dendritic lithium growth preferentially initiates at the remaining contact regions between the SSE and the current collector, ultimately leading to short‐circuiting. X‐ray tomography analysis visualizes electrochemically active lithium in yellow, revealing that after stripping corresponding to 98% of the total capacity, only about 6% of the initial contact area remains active—clear evidence of current crowding and uneven stripping. Therefore, ensuring uniform stripping to prevent localized lithium depletion is key to improving the performance and longevity of AFASSB systems. In this context, even when lithiophilic layers are introduced to the SSE‖anode interface to facilitate Li nucleation, they may not fully prevent direct contact between deposited Li and the SSE, leaving room for SSE decomposition, dendritic growth, and parasitic electronic pathways [56, 57, 58, 59, 60]. Thus, suppressing direct SSE‖Li contact and stabilizing the deposition front are also critical considerations in designing high‐performance anode‐free systems.

Figure 4c provides a mechanistic view of interfacial degradation during lithium stripping. During stripping, lithium vacancies can be redistributed into the bulk, which delays void accumulation at the interface at low current densities. As the stripping current increases, however, interfacial contact progressively deteriorates because voids accumulate more rapidly, leading to local current constriction during subsequent plating [61]. External pressure can partially mitigate this effect by improving interfacial conformity and maintaining SSE‖Li contact during cycling [62].

Stack pressure during cycling, as illustrated in Figure 4d, is a particularly critical factor influencing deposition uniformity in AFASSBs [52]. Even a slight collector tilt of approximately 0.4° induces a non‐uniform pressure distribution, which results in irregular lithium island growth. Therefore, maintaining uniform stack pressure across the SSE‖current collector interface is essential for homogeneous plating and stripping. Comparative studies at the pouch‐cell scale, as shown in Figure 4e, examined various pressure‐application methods, including rigid jigs, rubber layers, and isostatic configurations, and revealed that isostatic pressure yields the most uniform interfacial contact, leading to improved voltage profiles and enhanced cycling stability [53]. Nevertheless, its precise role in anode‐free configurations remains insufficiently understood. Temperature also strongly influences nucleation behavior, as depicted in Figure 4f. Lower temperatures slow lithium‐ion kinetics, increasing the number of nucleation sites [54]. In addition, some protective interlayer systems rely on multi‐step lithiation or conversion‐assisted deposition processes, in which different Li‐reactive phases sequentially form and influence Li nucleation and growth [63]. These observations further indicate that controlling nucleation pathways and interfacial contact is central to achieving homogeneous Li plating/stripping in AFASSBs.

### 2.3 Volume Changes

In battery systems, the components of a cell exhibit varying degrees of volumetric change that evolve dynamically during operation. In ASSBs, maintaining intimate interparticle contact is directly correlated with electrochemical performance. During lithium insertion and extraction within a rigid solid framework, interparticle contact can be lost, necessitating the application of external pressure to maintain structural integrity. For example, single‐crystal NCM811 cathodes can exhibit a unit‐cell volume contraction of about 4.15% upon delithiation, thereby reducing interparticle contact under low‐pressure solid‐state cell conditions [64]. Similarly, the theoretical volume change for LiFePO_4_ (LFP) cathodes is approximately 6.77% [65]. While conventional cathode materials generally exhibit comparable levels of volumetric variation, anode materials display much more pronounced differences depending on their chemical composition and lithiation mechanism. Graphite typically exhibits ∼10% expansion, whereas fully lithiated silicon can expand by up to 300%. Anode materials, unlike cathodes, often undergo larger volumetric changes depending on their composition and lithiation mechanism. Such extensive expansion and contraction can result in pronounced fluctuations in overall volume within all‐solid‐state cells, which, in turn, induce significant variations in internal pressure in fixed‐cell configurations. Consequently, these pressure fluctuations at the anode should be considered critical factors that directly influence the electrochemical performance and mechanical stability of solid‐state batteries. In anode‐free systems, deposition directly translates into volume increase, making uniform in‐plane deposition essential to minimize pore formation and vertical swelling.

**FIGURE 5:**
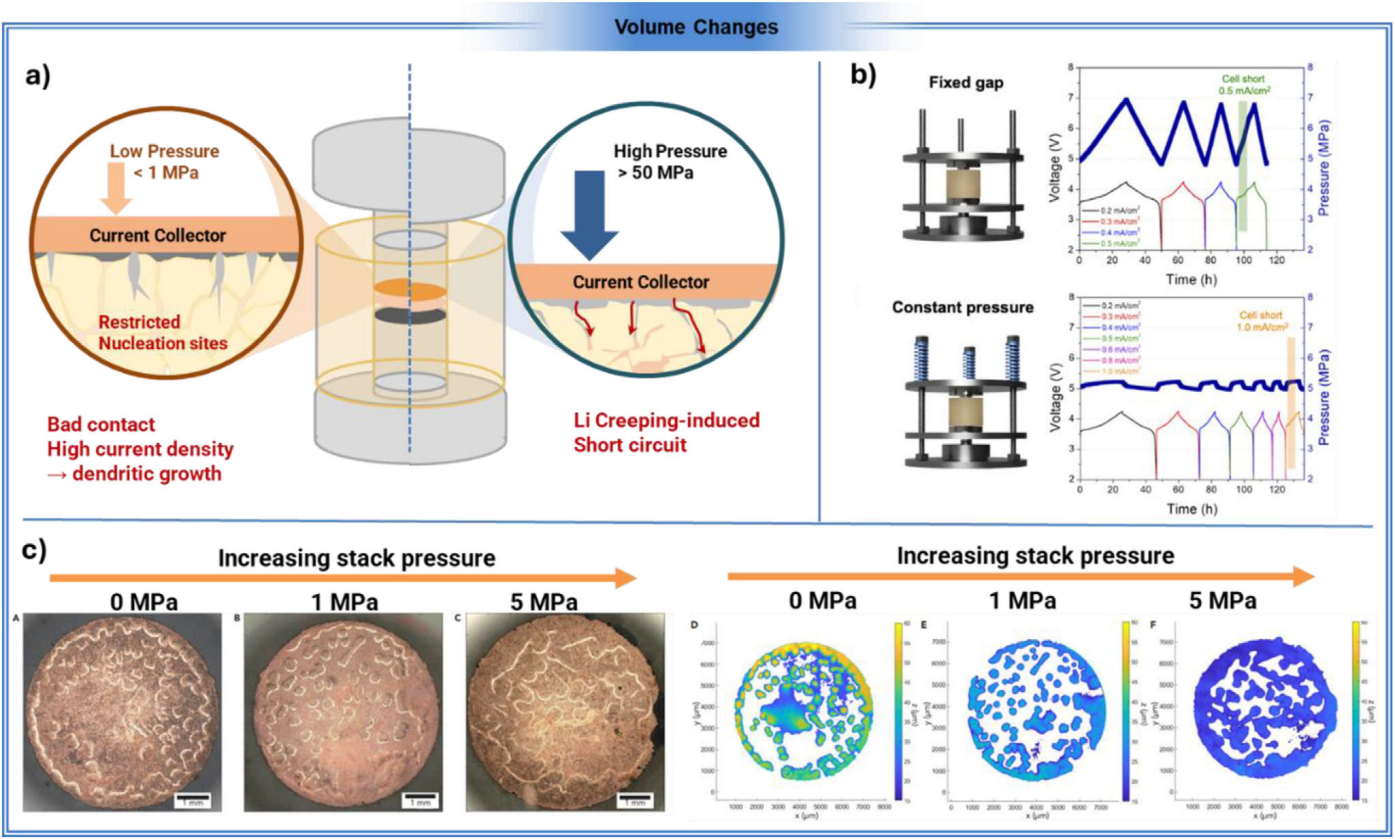
Influence of stack pressure on Li plating behavior and volume changes in solid‐state batteries. (a) Stack pressure effects in anode‐free cells employing sulfide SSEs: insufficient pressure causes poor interfacial contact between the SSE and current collector, restricting Li nucleation and promoting dendritic growth, whereas excessively high pressure limits lateral growth of Li and promotes creep penetration into the SSE. (b) Stack pressure mitigation in sulfide SSE‐based Li metal full‐cells: spring maintains constant pressure and interface during cycling, enhancing attainable current density. Reproduced with permission [66]. Copyright 2023, Elsevier. (c) Effect of stack pressure on plating uniformity in AFASSBs: higher stack pressures improve Li plating uniformity. In garnet‐type LLZO electrolytes, reduced SSE decomposition and distinct stack pressure effects were observed optically. Reproduced with permission [52]. Copyright 2022, Cell Press.

Stack pressure plays a decisive role in determining both the deposition morphology and overall volume evolution, as illustrated in Figure 5a. Conventional Li‐ion cells generally operate below 1 MPa, which is insufficient for sulfide‐based anode‐free systems, where poor interfacial contact between the solid electrolyte and current collector leads to localized current spikes and dendrite formation. Conversely, excessive pressure (>50 MPa) promotes lithium creep and can trigger short‐circuiting. Doux et al. reported that symmetric Li/Li cells with sulfide SSEs shortened after 48 h at 25 MPa at room temperature [67]. Although the critical pressure for lithium creep depends on factors such as electrolyte loading, areal capacity, and operating temperature, these findings underscore the necessity of precise pressure control in anode‐free configurations.

To mitigate such issues, mechanical compliance introduced into the cell fixture, as shown in Figure 5b, can effectively accommodate changes in internal volume. Incorporating springs into triaxial jigs improved cycling stability and increased the critical current density in Li‐metal cells, and similar approaches are expected to benefit anode‐free systems as well. Moreover, in situ optical observations shown in Figure 5c revealed that at low stack pressures (< 5 MPa), lithium deposits formed taller, irregular islands on Cu surfaces, clearly demonstrating the strong dependence of Li‐plating morphology on applied pressure [52]. These results emphasize the importance of designing cell architectures that can accommodate internal volume fluctuations.

## 3 Key Strategies for High‐Performance Anode‐Free All‐Solid‐State Batteries

In the previous section, we discussed the key degradation factors in sulfide‐based AFASSBs, including (1) interfacial instability of sulfide‐based SSEs, (2) non‐uniform Li plating/stripping, and (3) volume changes during repeated cycling. The limitations above lead to rapid capacity fading and poor long‐term cycling performance due to the inherently low Coulombic Efficiency (CE), which worsens with repeated cycling in the absence of excess Li. A variety of strategies have been investigated to overcome these critical challenges. Among the various approaches proposed to improve the electrochemical performance of AFASSBs, we categorize them into the following groups for clarity and systematic discussion: (1) interlayer engineering, including carbon‐based interlayers, conversion‐type interlayers, and dual‐metal seed layers; (2) pressure‐regulating buffer layers, such as carbon felt, Mg/MXene dual thin‐film layer, elastomeric silicon interlayers, and 3D Li host layers; (3) other methods, including the implementation of sacrificial cathodes, modification of SSEs, and cell assembly and cycle protocol.

### 3.1 Interlayer Engineering

To overcome the various limitations of AFASSBs discussed above, a lithiophilic metal seed coating layer on the current collector has been proposed [56, 57, 68, 69, 70, 71]. With these layers on the current collector, the nucleation energy barrier and the associated overpotential could be reduced, homogenizing the initial Li coverage. This interlayer concurrently strengthens chemo‐mechanical coupling at the SSE‖current collector interface, enhancing charge–discharge reversibility and enabling the development of high‐performance AFASSBs.

**FIGURE 6:**
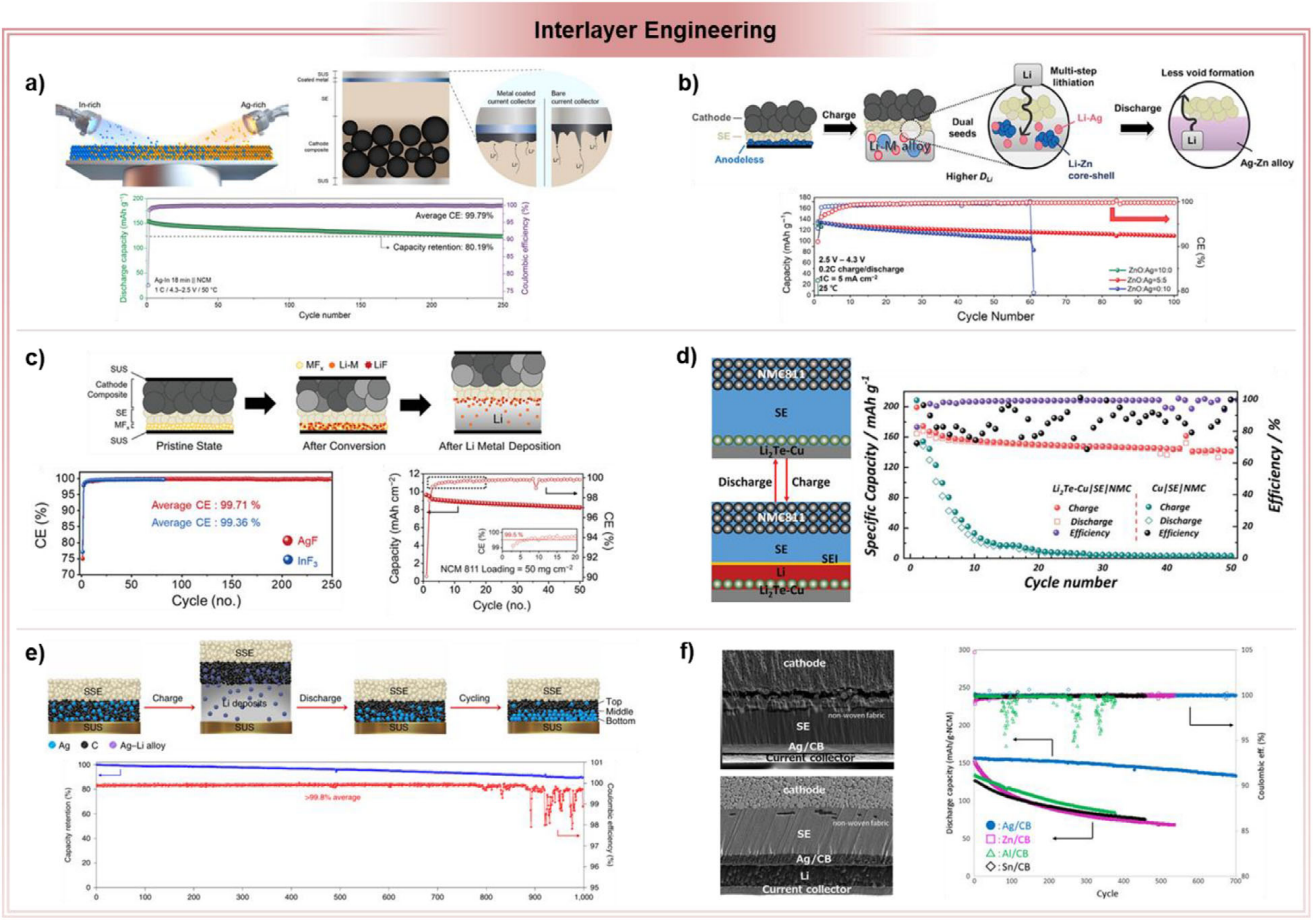
Various interlayer engineering strategies to enhance cycle performance of AFASSBs: (a) Coating multi‐metals using the CCS sputtering method. Schematic diagram of a full‐cell with the bare and metal‐coated current collector. Cycle performance of the Ag‐In‖SSE‖NMC full‐cell. Reproduced with permission [68]. Copyright 2024, Elsevier. (b) Schematic illustration of Li plating/stripping process on the AgZnO electrode. Cycle performance of AgZnO, ZnO, and Ag‐based full‐cells at 25°C. Reproduced with permission [69]. Copyright 2024, The Authors. (c) Schematic illustration of the conversion reaction followed by Li metal deposition during cycling. Cycle performance of AgF‐ and InF3‐based ASSB half‐cells. Cycle performance of AgF‐based full‐cells at 25°C. Reproduced with permission [57]. Copyright 2022, Wiley‐VCH. (d) Schematic illustration of the working principle of the conversion‐type interlayer in AFASSBs. Cycle performance of Li2Te‖SSE‖NMC and Cu‖SSE‖NMC. Reproduced with permission [56]. Copyright 2023, Wiley‐VCH. (e) Schematic illustration of Li plating/stripping behavior through an Ag‐C nanocomposite interlayer (top). Cycle performance of Ag/C‖SSE‖NMC prototype pouch cell (bottom) tested at 60°C. Reproduced with permission [70]. Copyright 2020, Springer Nature. (f) Cross‐sectional SEM images of ASSBs with Ag/CB‐based anode after first charge (top) and discharge (bottom). Cycle performance of ASSB with different metal/CB‐based anodes. Reproduced with permission [71]. Copyright 2021, The Authors.

For instance, Lee et al. reported a doped lithiophilic metal seed coating strategy (Figure 6a) [68]. After optimizing the Ag‐In composition, they fabricated an In‐doped Ag coating on the current collector via continuous composition spread (CCS) sputtering, achieving synergistic improvements in interfacial nucleation and stability, where the CCS method was used to explore compositions containing two or more different elements [72]. Ag has been widely researched for years due to its ability to stabilize the interface, leading to a long cycle life, and it has been implemented to promote solid‐solution reactions with Li. Analogous to the passivating role of carbon layers in Ag‐C nanocomposites [70], Lee et al. underscored the need for complementary materials to passivate the interface reaction of SSEs. To this end, a small fraction of In was introduced into Ag. Indium, widely employed in Li‐In alloy anodes for ASSB studies, offers a low energy barrier for Li diffusion and chemical inertness, which together mitigate interfacial reactions and suppress SSE decomposition [73]. The resultant Ag‐In coated current collector combines Ag‐driven nucleation/growth facilitation with In‐enabled interfacial stabilization and faster Li kinetics, yielding an Ag‐In‖NCM full‐cell that retains 80.2% of its capacity after 250 cycles.

Later, Sohn et al. reported a dual‐seed‐based protective layer for AFASSBs operated at room temperature, implementing zinc oxide (ZnO) and Ag (Figure 6b) [69]. ZnO is inexpensive to synthesize and highly reactive with Li, enabling conversion followed by alloying. Ag, by contrast, serves as a lithiophilic nucleation seed. The electrochemical performance of the AgZnO, Ag, and ZnO anode‐free electrodes was evaluated by testing half‐cells at room temperature to assess the superiority of the dual‐seed strategy. The dual‐seed AgZnO half‐cell delivered the highest initial Coulombic Efficiency (ICE) of 92.24% and sustained stable cycling without short‐circuiting for 260 cycles with an average CE of 99.27%. This superior behavior is attributed to a multi‐step lithiation mechanism operative during plating/stripping. Upon initial Li intake, ZnO undergoes a conversion reaction to form Zn, followed by Zn‐Li alloying. In parallel, Ag forms Li‐Ag alloys. As the potential decreases, Li‐Zn and Li‐Ag phases nucleate concurrently, with Li‐Ag alloy formation becoming predominant. Consequently, a well‐mixed AgZnO network offers multiple reaction pathways across a broader potential range, whereas single‐seed designs are restricted to a narrower range [63]. Leveraging these effects, dual‐seed AgZnO full‐cells achieved a capacity retention of 80.8% after 100 cycles at 1 mA cm^−2^ and 3 mAh cm^−2^ at room temperature.

While lithiophilic metal seed coating layers promote uniform Li plating, they may not always prevent direct contact between plated Li and the SSE, leaving room for SSE decomposition, dendritic growth, and parasitic electronic pathways. As a complementary route, conversion‐type interlayers have been introduced to regulate interfacial reactions while maintaining favorable Li deposition behavior [56, 57].

Room‐temperature operable AFASSBs incorporating a conversion‐type metal fluoride interlayer were first demonstrated by Lee et al. [57]. (Figure 6c) During the initial cycles, metal fluorides undergo a conversion reaction with Li to yield electronically insulating LiF and a metal (M) that subsequently participates in alloying. Among several candidates, AgF was selected as the Li‐Ag solid‐solution reaction is kinetically favorable. AgF‐based half‐cell delivered an average CE of 99.7% over 250 cycles. In contrast, metal fluorides exhibiting relatively sluggish intermetallic reactions showed rapid capacity decay. The advantages of in situ conversion of AgF are two‐fold: (1) the resulting metallic Ag domains act as nucleation seeds, enabling spatially homogeneous and facile Li deposition; and (2) the concurrently formed electronically insulating LiF passivates the SSE‖plated Li interface and thereby suppressing SSE reduction during charging. Consequently, full‐cells incorporating the AgF‐based anode‐free electrode achieved an ICE of 90.1%, 85.4% capacity retention after 50 cycles at room temperature, and an average CE of 99.7%.

Wang et al. later reported a Li‐activated telluride coating on a Cu current collector (Figure 6d) [56]. Lithium telluride (Li_2_Te) was chosen because it is lithiophilic, kinetically/thermodynamically stable, and fabrication‐friendly, requiring only conventional vacuum deposition and thus potentially scalable. The coating was formed by in situ Li activation of a copper telluride (Cu_2_Te) intermetallic on Cu via an irreversible conversion reaction. The resulting stable, lithiophilic Li_2_Te surface improves Li wetting and facilitates electrodeposition and dissolution, yielding a substantially higher critical current density (CCD) than bare Cu. The Li_2_Te‐Cu electrode delivered a CE of 99.70% in half‐cells, compared with 98.47% for the bare Cu. This gap is attributed to a combination of SEI growth from the reaction between SSE and plated Li and the formation of electrochemically inactive dead metal on the current collector surface. In full‐cell testing, a Cu_2_Te‖SSE‖NCM configuration achieved 80% capacity retention after 50 cycles.

Beyond metal seed coating layers and conversion‐type interlayers, carbon‐based interlayers have also been reported to improve the performance of AFASSBs [70, 71]. For instance, Lee et al. reported AFASSBs comprising an Ag‐C nanocomposite layer as the anode instead of Li metal (Figure 6e) [70]. In this design, a carbon interlayer, rather than a conventional 3D Li host, primarily functions as a separator to maintain interfacial stability, while Ag facilitates Li deposition. This configuration enhances SSE durability and prevents Li penetration through it. In addition, implementing warm isostatic pressing (WIP), a technique that applies isostatic pressure to promote intimate contact and heat the interface to melt it, further improves effective electrode‐electrolyte contact [74]. Consequently, a prototype pouch cell (0.6 Ah) delivered a higher energy density (>900 Wh L^−1^), a stable CE (>99.8%), and a long cycle life (1000 cycles). The transport mechanism of Ag, however, remains incompletely understood, and ongoing studies aim to elucidate this mechanism.

Suzuki et al. reported an overcharge‐type anode in sulfide‐type ASSBs, clarifying the effect of carbon black (CB) (Figure 6f) [71]. ASSBs employing a graphite‐based anode or an anode without a carbon layer exhibited rapid short‐circuiting at early stages. In contrast, the CB‐based anode cycled without short‐circuiting and retained 87% of its capacity over 150 cycles. These results suggest that a carbon‐containing interlayer can provide a more favorable deposition environment and contribute to more stable cycling behavior, although the detailed deposition pathway still requires further verification.

#### 3.1.1 Mechanism of Li Transport in Carbon‐Based Interlayers

As mentioned in the previous section, the development of AFASSBs employing an Ag‐C nanocomposite layer, reported by Lee et al., has brought a significant paradigm shift in this research field [70]. However, several questions remain unresolved. It is still unclear how Ag facilitates uniform current distribution while suppressing Li dendrite formation, why Ag tends to migrate toward the current collector, why Li deposition occurs exclusively at the SSE‖current collector interface, and why other metallic interlayers fail to deliver comparable cycle stability [1]. Consequently, many researchers have extensively addressed these fundamental questions through theoretical and experimental investigations [75, 76, 77]. Further performance enhancement strategies must be developed to achieve commercially viable AFASSBs, which fundamentally requires a thorough understanding of the mechanisms described in this section.

**FIGURE 7:**
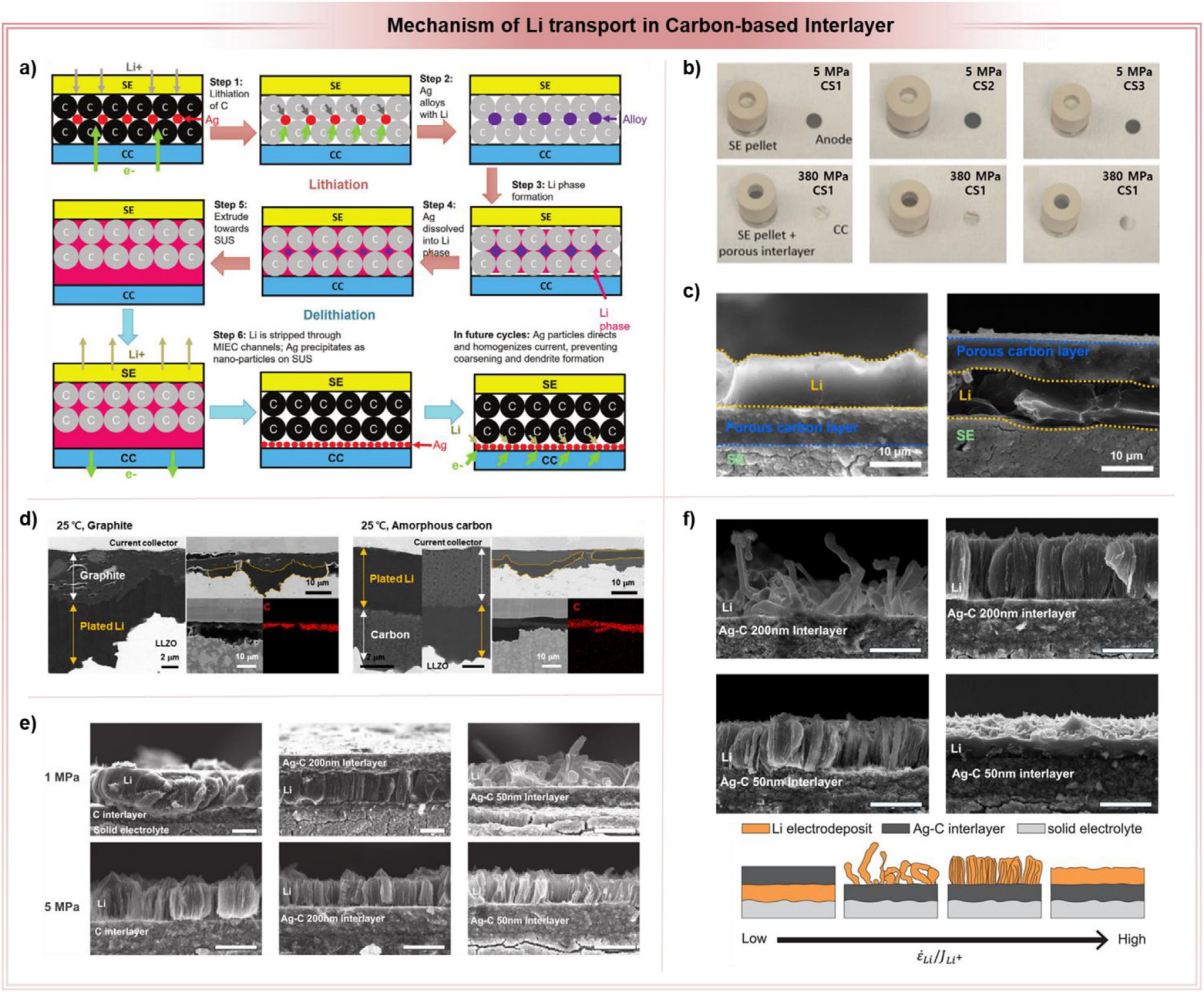
Mechanisms of Li transport in carbon‐based interlayer: (a) Microscopic lithiation‐delithiation mechanism of Ag/C interlayer. Reproduced with permission [78]. Copyright 2024, The Authors. (b) Images from peel‐off tests of the anode‖SSE pellet assembly following the SSE‐porous layer contact formation at 5 MPa (top) and 380 MPa (bottom). Reproduced with permission [75]. Copyright 2022, The Royal Society of Chemistry. (c) Cross‐sectional SEM images presenting Li‐plated composite anodes prepared with porous interlayer‖SSE contact at 380 MPa (left) and amorphous carbon (right) layers at 0.5 mA cm^−2^ to a capacity of 3 mAh cm^−2^. Reproduced with permission [75]. Copyright 2022, The Royal Society of Chemistry. (d) Cross‐sectional SEM and EDS images of LLZO SSE‖anode interfaces showing Li plating through graphite (left) and amorphous carbon (right) layers at 25°C. Reproduced with permission [77]. Copyright 2022, The Authors. (e) Cross‐sectional SEM images of interlayers (carbon, Ag‐C (200 nm), and Ag‐C (50 nm), respectively) charged at 0.5–2 mAh cm^−2^ under 1 and 5 MPa. Reproduced with permission [76]. Copyright 2024, The Authors. (f) Cross‐sectional SEM images of Li‐plated Ag‐C (200 nm) interlayer (top) and Ag‐C (50 nm) interlayers (middle) after charging with a current density of 0.25 (left) and 0.1 mA cm^−2^ (right). Schematic illustrations of Li electrodeposits morphology varying with JLi0, Creep / JLi+, Reduction. Reproduced with permission [76]. Copyright 2024, The Authors.

Xie et al. sought to elucidate the underlying mechanisms within the Ag/C interlayer through first‐principles thermodynamic calculations and continuum modeling [78]. Their computational analysis revealed why Ag performs better than other metals when used in the Ag/C interlayer. The cascading process of lithiation and delithiation along the Ag/C interlayer is shown in Figure 7a. At the initial stage of lithiation, the amorphous carbon, acting as a mixed ionic‐electronic conductor (MIEC), becomes saturated with Li. Subsequently, Ag undergoes an alloying reaction with Li, forming a solid‐solution phase. As lithiation proceeds, the expansion of the Li‐Ag alloy separates the interlayer from the current collector interface, leading to extrusion of the alloy toward the current collector. Meanwhile, the remaining Ag and Ag‐rich phases dissolve into the solid solution and flow plastically in the same direction. During delithiation, Li is extracted from the solid solution, promoting Ag precipitation. The uniformly distributed Ag nanoparticles on the current collector surface play a crucial role in preventing Li current concentration at surface tips, mitigating surface coarsening, and suppressing dendritic growth during subsequent cycling. Other metals (e.g., Sn, Zn, Al, and Ni) are less effective than Ag, primarily because of their unfavorable lithiation properties. Their lithiation potentials tend to become negative at relatively high overpotentials or the premature termination of the lithiation process before sufficient Li uptake occurs. As a result, they cannot undergo significant volume expansion or effectively migrate toward the current collector as Ag does.

Meanwhile, Park et al. aimed to elucidate why Li plating occurs at the interface between the interlayer and the current collector in AFASSBs employing Ag‐C nanoparticles [75]. They proposed that Li plating preferentially occurs at the porous interlayer‖current collector interface rather than at the SSE‖porous interlayer interface when the adhesion strength between the interlayer and SSE exceeds that between the interlayer and the current collector. To verify this hypothesis, they compared the adhesion energies at each interface using peel tests, in which fabrication pressures of 5 and 380 MPa were applied to form interfacial contacts between the SSE and the porous interlayer. When a pressure of 5 MPa was applied, the anode layer detached entirely from the SSE pellet, indicating that the adhesion energy between the SSE and the porous interlayer was weaker than between the porous interlayer and the current collector. In contrast, under a pressure of 380 MPa, delamination occurred only between the current collector and the porous interlayer, while the porous interlayer remained attached to the SSE surface, confirming that the SSE‖porous interlayer interface possessed stronger adhesion energy (Figure 7b).

To further substantiate these observations, Li plating was conducted at a current density of 0.5 mA cm^−2^ up to a capacity of 3 mAh cm^−2^, and cross‐sectional SEM images were obtained for cells fabricated under both 5 and 380 MPa conditions (Figure 7c). The experimental results were consistent with their hypothesis: under 380 MPa, Li metal deposited at the interface between the current collector and the porous interlayer, whereas under 5 MPa, Li metal formed at the interface between the SSE and the porous interlayer. Based on these findings, the authors concluded that Li preferentially deposits at regions within the composite anode where the Gibbs free energy decreases most significantly. Additionally, they proposed that, beyond this thermodynamic factor, kinetic parameters such as pore size, interlayer composition, temperature, and lithiophilicity also play essential roles in facilitating Li migration and deposition.

Kim et al. conducted a comparative study of amorphous carbon and graphite interlayers [77]. They assembled asymmetric half‐cells of Li metal, an LLZO pellet, and a current collector to probe Li plating through carbon layers. After electrochemical depositions at 100°C, 60°C, and 25°C, they examined Li distribution using cross‐sectional SEM and EDS. At 100°C, where kinetic barriers are sufficiently low, Li preferentially plated at the interface between the interlayer and the current collector, consistent with the trend reported by Suzuki et al. [71]. However, lowering the temperature disrupted this preference. SEM and EDS images revealed that Li deposition occurs at the interlayer‖current collector interface and at the interface between the SSE and the interlayer, making this behavior more prominent for graphite (Figure 7d).

These observations indicate a competition between thermodynamically favored nucleation sites and Li transport kinetics at lower temperatures. When transport through the interlayer is sluggish, Li deposition becomes randomized despite the energetic preference, because plating in an isolated region is kinetically hindered. Results at 60°C support this interpretation: relative to 25°C, improved kinetics enable more uniform plating. With amorphous carbon, Li readily deposits at the interlayer‖current collector interface at 60°C, underscoring the temperature sensitivity of Li plating behavior. In contrast, graphite still shows partial deposition at the interlayer‖SSE interface at 60°C, which the authors attribute to the two‐dimensional (2D) character of Li diffusion in graphite. Composed of stacked graphene basal planes, the graphitic framework dictates that Li diffusion proceeds via intercalation and deintercalation [79]. This pathway is anisotropic, inherently hindering vertical Li transport through the interlayer. Conversely, the highly disordered structure of amorphous carbon, coupled with transport mechanisms governed by adsorption, desorption, and Li‐cluster accommodation within nanopores, enables isotropic Li transport [80]. Consequently, these features fundamentally facilitate vertical Li transport through the interlayer. Beyond temperature, the intrinsic Li diffusion characteristics of each carbon interlayer therefore play a decisive role in determining where Li plates.

Park et al. investigated Li transport and electrochemical deposition behavior within Ag‐C porous interlayers in AFASSBs [76]. They revealed that electro‐chemo‐mechanics coupling significantly affects Li deposition behavior and overall cell performance. In their work, the strain rate for Li metal (*ε_Li_*),[A Model for Boundary Diffusion Controlled Creep in Polycrystalline Materials] the creep‐induced Li metal flux (*J_Li_^0^, _Creep_*), and the Li accumulation flux into pores from Li‐ion reduction (*J_Li_^+^, _Reduction_*) were defined as following Equations (1), (2), and (3), respectively.

(1) $$\varepsilon_{\mathrm{Li}}=K\left(\delta_{s}D_{s}\Omega /D^{3}K_{B}T\right)\sigma$$

(2) $$J_{Li^{0},\mathrm{Creep}}=r\cdot \varepsilon_{\mathrm{Li}}$$

(3) $$J_{Li^{+},\;\mathrm{Reduction}}=I\cdot \Phi^{-1}\cdot F^{-1}\cdot Vm^{-1}$$

In the strain rate for Li metal, *K* represents a dimensionless constant, *δ_s_* is the nominal surface layer thickness, *D_s_* is the surface diffusivity of Li, *Ω* is the atomic volume of Li, *K_B_* is the Boltzmann constant, *T* is the absolute temperature, *σ* is the applied pressure, and *D* is the average grain diameter. The creep Li flux was derived from the strain‐rate equation, where *r* corresponds to the pore diameter. In the equation for the Li accumulation flux into the pore, *I* is the current density, *Φ* is the porosity of the interlayer, *F* is the Faraday constant, and *V_m_* is the molar density of Li. Li can migrate through the interlayer when the creep Li flux exceeds the Li accumulation flux. Conversely, when the Li accumulation flux dominates, pores become blocked by Li, leading to Li accumulation at the SSE surface and, eventually, interface splitting.

To examine the effect of the composition of the interlayer and applied internal pressure, three types of interlayers were tested: a pure carbon layer, a composite of carbon black and 200 nm Ag nanoparticles (Ag‐C (200 nm) interlayer), and a composite of carbon black and 50 nm Ag nanoparticles (Ag‐C (50 nm) interlayer) (Figure 7e). Under low‐pressure (1 MPa) conditions, the pure carbon interlayer enabled Li accumulation within the porous network, leading to local delamination and cracking. Some Li penetrated through these cracks and deposited on the current collector. Incorporating Ag nanoparticles would facilitate Li movement and induce uniform Li plating [59, 60, 81]. However, in the Ag‐C (200 nm) interlayer, Li migration toward the current collector was not effectively achieved; instead, Li primarily accumulated at the SSE‖interlayer interface, suggesting that the particle size of Ag strongly influences Li diffusion behavior. When the Ag nanoparticle size was reduced from 200 to 50 nm, Li could diffuse more efficiently through the interlayer and deposit at the current collector interface. Nevertheless, the non‐uniform distribution of Ag nanoparticles led to a concentrated ionic flux, which could trigger dendritic growth. When the fabrication pressure was increased to 5 MPa, all interlayers exhibited uniform Li deposition near the current collector, indicating that the creep Li flux exceeds the Li accumulation rate under relatively high‐pressure conditions. The effect of pore size evolution was also investigated. In the Ag‐C (200 nm) interlayer, the volume change associated with the Ag‐Li alloying reaction led to significant local stress accumulation near Ag particles, producing larger pores and structural deformation of the particle stacking. These effects hindered the creep Li flux and caused incomplete alloying, leading to Li accumulation at the SSE‖interlayer interface. In contrast, the Ag‐C (50 nm) interlayer showed less stress accumulation and more complete alloying, allowing for effective pore filling and Li transport through the interlayer to the current collector. The Ag‐C (50 nm) interlayer exhibited the highest Li diffusion coefficient among the three interlayers.

The influence of current density on Li plating morphology was also evaluated (Figure 7f). For the Ag‐C (200 nm) interlayer under low‐pressure conditions, Li was plated at the SSE‖interlayer interface when the current density was 0.5 mA cm^−2^. At 0.25 mA cm^−2^, Li deposition occurred at the interlayer‖current collector interface in whisker‐like morphology, whereas at 0.1 mA cm^−2^, a compact, uniform Li metal electrode formed. For the Ag‐C (50 nm) interlayer, Li consistently deposited at the current collector across all current densities; however, its morphology varied, showing dendritic growth at 0.5 mA cm^−2^, fiber‐clustered structures at 0.25 mA cm^−2^, and dense, smooth layers at 0.1 mA cm^−2^. These results demonstrate that Li transport occurs through the interlayer when the creep Li flux is much faster than the accumulation flux. In contrast, when the accumulation flux dominates, numerous pores become blocked, leading to localized Li at the SSE surface.

In summary, the Ag‐C nanocomposite interlayer, first introduced by Lee et al., has attracted significant attention for its role as an MIEC, enabling unique Li transport behavior [70]. Section 3.1.1 highlights studies that have provided in‐depth insights into the Li transport mechanisms in such systems. Although a definitive mechanism has not yet been fully established, it is generally accepted that Li deposition at the interlayer‖current collector interface arises from the synergistic interaction between amorphous carbon and Ag nanocomposite. During the initial charge, Li is electrochemically deposited onto the interlayer and partially consumed through irreversible reactions with carbon. This interaction leads to the formation of a Li‐containing MIEC layer, which subsequently serves as a nucleation site for further Li growth. In the early stages of nucleation and growth, Li atoms or ions diffuse along the surface, contributing to uniform Li deposition [82]. Under stack pressure, metallic Li is further transported within the MIEC layer via diffusional Coble creep [83]. Concurrently, alloying between Ag and Li occurs within the layer, leading to the formation of various Ag‐Li alloy phases. The resulting phase gradient within the layer provides energetically favorable pathways that facilitate redistribution [78]. In addition, interfacial adhesion at the SSE‖interlayer and interlayer‖current collector interfaces plays an important role in governing Li deposition at the interlayer‖current collector interface [75, 84]. Therefore, Li transport and deposition behavior in the Ag‐C interlayer system cannot be attributed to a single mechanism such as surface diffusion, alloy phase redistribution, or MIEC network alone, but rather arises from the complex interplay among these processes. Furthermore, factors such as the size of Ag nanoparticles, porosity of the MIEC layer, current density, and temperature also significantly influence Li transport in AFASSBs.

### 3.2 Pressure‐Regulating Buffer Layers

Beyond the heterogeneous Li plating caused by a high nucleation energy barrier, Li protrusions, and electrically isolated ‘dead Li’, limitations also arise during stripping as previously mentioned in Section 2.2 [18, 19, 47, 85]. Accordingly, many groups have reported various buffer layers to remediate these failure modes [83, 86, 87, 88, 89].

**FIGURE 8:**
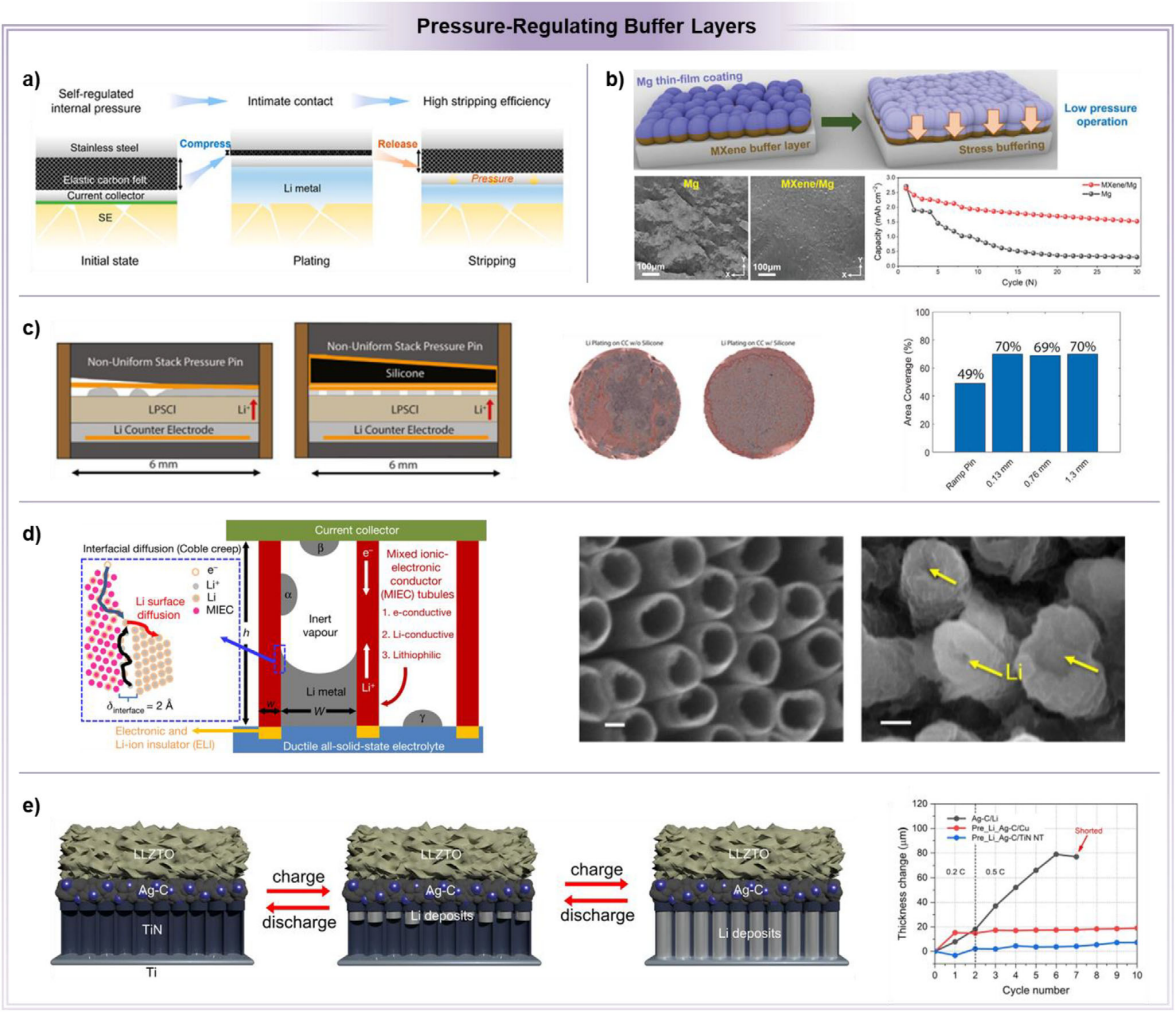
Strategies concerning pressure‐regulating buffer layers to enhance cycle performance of AFASSBs: (a) Schematic illustration of the working mechanism of the elastic carbon felt layer during cycling. Reproduced with permission [86]. Copyright 2023, American Chemical Society. (b) Schematic illustration of lithiation on MXene/Mg double layer at low operating pressure. XRM images of both Mg anode‐free electrode (left) and MXene/Mg anode‐free electrode (right): 2D images of anode‖SSE interface. Cycle performance of full‐cells using Mg anode‐free electrode and MXene/Mg anode‐free electrode. Reproduced with permission [87]. Copyright 2023, Wiley‐VCH. (c) Cell stack with non‐uniform stack pressure inducing pin with/without silicon interlayer (left). An optical image presenting the difference in Li plating behavior on the current collector with/without a silicon interlayer (middle). Plated Li coverage with various thicknesses of silicon interlayer (right). Reproduced with permission [88]. Copyright 2024, Elsevier. (d) Creep‐enabled Li plating/stripping in a 3D Li host: Schematic of the process in MIEC tubules (right) and field‐emission SEM (FE‐SEM) images of Li metal deposited within the tubule (left). Reproduced with permission [83]. Copyright 2020, Springer Nature. (e) Schematic illustration of charge/discharge processes in a garnet‐type SSE‐based AFASSB incorporating TiN NTs (right), and thickness change profiles of various pouch‐type full‐cells as a function of cycle number (left). Reproduced with permission [89]. Copyright 2024, Springer Nature.

For instance, Cao et al. reported a conductive, elastic carbon felt layer to address contact loss during stripping that drives rapid capacity fading and low CE (Figure 8a) [86]. Placed between the current collector and a pressing pillar, the carbon felt (1) buffers volume expansion during Li plating by compressing under rising internal pressure, thereby reducing the propensity for Li penetration into the SSE, and (2) self‐adjusts pressure during Li stripping as it elastically relaxes, preserving intimate Li‖SSE contact. The carbon felt suppresses dead Li accumulation and the attendant CE penalty by mitigating interfacial void formation. Consequently, the ICE increased from 58.4% to 83.7%, and cycling stability improved by more than an order of magnitude.

In the same year, Oh et al. reported the first AFASSBs that operate at room temperature under low stack pressure (Figure 8b) [87]. Cycling AFASSB cells under this regime is difficult because Li plating/stripping kinetics are sluggish, and interfacial voids readily form. They introduced a bilayer buffer comprising Mg and the MXene Ti_3_C_2_T_x_ to address both issues. Mg was chosen as a lithiophilic, alloying‐forming metal; among several candidates (Mg, Ag, Au, and Zn), it delivered the highest ICE and a cycle life exceeding 1100 h. The Ti_3_C_2_T_x_ MXene, a conductive, 2D ceramic with high ductility, was added to enable low‐pressure operation, thereby relieving stress from volume changes and maintaining intimate interfacial contact during Li plating/stripping [90, 91]. As a result, even at 2 MPa and room temperature, the bilayer suppressed void formation, preserved the intimate interface, and minimized capacity loss and overpotential growth.

Later, Thorpe et al. addressed the persistent issue of non‐uniform external stack pressure in AFASSBs by inserting an elastomeric silicon interlayer behind the current collector (Figure 8c) [88]. Because intimate SSE‖current collector contact is crucial, electrode roughness and angular misalignment of the pressing plates often produce uneven pressure and voids. Using a ramp pin geometry to deliberately induce non‐uniform loading, the silicon layer increased Li plating coverage from 49% to 70%, resulting in a more uniform deposit morphology. With a stainless steel mesh in place, the interlayer disrupted the periodic plating pattern, distributing nucleation sites more randomly and evenly, thereby raising CE from 89% to 94%. Overall, the elastomeric buffer compensates pressure gradients, markedly improving the uniformity and reversibility of Li plating/stripping under practical, imperfect loading conditions.

Li metal has an atomic volume of 21.6 Å^3^/atom = 0.135 eV/GPa. Thus, an overpotential of −0.135 V can generate hydrostatic stresses on the GPa scale, which are transmitted to the surrounding solid frameworks [92]. If the electrochemically induced mechanical stress is not relieved, Li dendrites can initiate cracks along grain boundaries or within the SSE lattice, continue to propagate during cycling, and ultimately cause short‐circuiting. Beyond 2D interlayers, Chen et al. addressed this persistent chemo‐mechanical and interfacial instability in AFASSBs by introducing a 3D MIEC as a Li host, engineered to serve as both an electronic and an ionic conductor and to exhibit lithiophilicity (Figure 8d) [83]. Using in situ TEM, they quantitatively identified diffusional Coble creep along the MIEC‖Li interface as the mechanism governing Li transport. During stripping, Li continued to be removed even when a void plug formed at the Li‖SSE contact, indicating that Li is extracted along the surface. In full‐cell tests, pre‐depositing only 1× excess Li within the MIEC tubules yielded lower overpotentials, higher discharge capacities, and improved CE, with minimal degradation over more than 50 cycles. Furthermore, the Li‖MIEC composite anode achieved a gravimetric capacity of ∼900 mAh g^−1^. These results demonstrate that a 3D Li host architecture can effectively mitigate pressure‐induced stress and chemical interfacial instabilities in AFASSBs.

Later, Kim et al. introduced titanium nitride nanotubes (TiN NTs) with MIEC properties, combined with an Ag‐C interlayer, to mitigate performance degradation arising from internal strain and volume changes during Li plating/stripping [89]. As in the previously discussed studies on interfacial diffusional creep, the TiN NTs effectively accommodated Li by surface diffusional creep. Reduced Li ions penetrated the free internal volume of the TiN NTs, where interfacial diffusion facilitated uniform Li deposition, thereby efficiently relaxing volume changes. The Ag‐C interlayer ensured intimate interfacial contact between Li_6.4_La_3_Zr_1.7_Ta_0.3_O_12_ SSE and the TiN NTs layer while rapidly transferring reduced Li metal into the TiN NTs, thereby promoting homogeneous Li plating. As illustrated in Figure 8e, the Ag‐C‐coated TiN NTs structure enabled uniform Li deposition within the free volume of the 3D Li host during repeated Li plating/stripping, effectively compensating for volume expansion. In contrast, in the conventional AFASSB configuration, Li deposition occurred primarily at the Ag‐C/Cu interface, leading to localized stress accumulation. Three pouch‐type full cells were assembled to compare dimensional changes during charging: Ag‐C/Li, pre‐lithiated Ag‐C/Cu, and pre‐lithiated Ag‐C/TiN NTs. The pre‐lithiated Ag‐C/TiN NTs cell suppressed volume expansion by approximately 85% relative to the other two cells. Moreover, thickness monitoring over extended cycling revealed that the TiN NT‐integrated cell exhibited only a minimal average change of 3.5 µm, demonstrating excellent buffering capability against volume variation. Overall, the authors successfully developed a TiN NT‐based anode architecture that accommodates Li volume change via a diffusion‐creep mechanism. The synergistic combination with the Ag‐C interlayer enabled uniform Li transport and deposition, offering a promising platform for realizing high‐energy‐density, long‐lifetime AFASSBs employing garnet‐type SSEs. Although this design was not based on sulfide‐based SSEs, the underlying concept could be readily extended to sulfide‐based AFASSBs, provided that suitable chemical and electrochemical compatibility is ensured.

### 3.3 Other Methods

Beyond interlayer engineering and the incorporation of pressure‐regulating buffer layers (as discussed in Sections 3.1 and 3.2, respectively) to address factors that can lead to performance degradation in AFASSBs, additional strategies have emerged. Section 3.3 presents these additional strategies for performance enhancement and is further divided into three distinct approaches. Section 3.3.1 focuses on the use of a sacrificial cathode additive to compensate for the limited Li reservoir, thereby mitigating the intrinsic issue of irreversible Li loss in AFASSBs. Section 3.3.2 introduces modifications to SSEs to stabilize interfacial chemistry and alleviate non‐uniform Li plating/stripping. Finally, Section 3.3.3 highlights strategies that enhance cell performance without introducing additional cell components, instead relying on optimized cell assembly and cycle conditions tailored for AFASSBs [49, 66, 93, 94, 95, 96, 97, 98, 99, 100]

#### 3.3.1 Implementation of Sacrificial Cathode Additives

**FIGURE 9:**
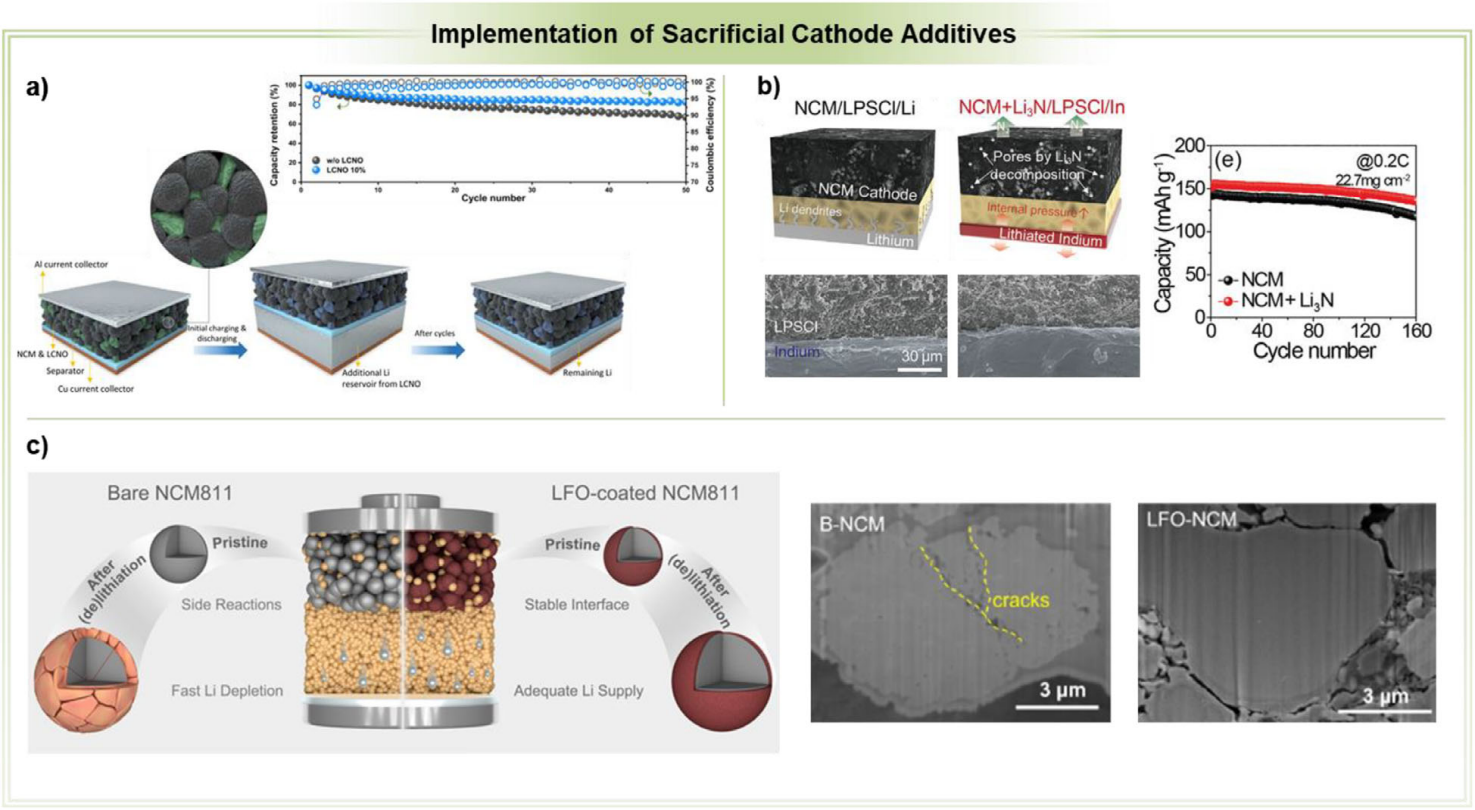
Representative strategies using sacrificial cathode additives to enhance cycle performance of AFASSB: (a) Schematic illustration of AFASSB with LCNO sacrificial additives during cycling. Cycle performance of AFASSB with/without LCNO. Reproduced with permission [93]. Copyright 2023, Elsevier. (b) Comparative schematic illustration of ASSBs with Li metal (top left) and In metal with Li_3_N sacrificial cathode (top right) during charging. Highly magnified cross‐sectional SEM image of LPSCl‖In interfaces of NCM+Li_3_N cells at the pristine state (bottom left) and the 50th cycle (bottom right). Cycle performance comparison between NCM and NCM+Li_3_N cells. Reproduced with permission [94]. Copyright 2021, Wiley‐VCH. (c) Schematic illustration comparing interfacial instability between bare NCM811 and LFO‐coated NCM811. Cross‐sectional SEM images of bare NCM and LFO‐coated NCM811 after 100 cycles. Reproduced with permission [95]. Copyright 2024, Wiley‐VCH GmbH.

As noted earlier, in AFASSBs, unlike all‐solid‐state Li metal batteries with excess Li inventory, the Li reservoir depends solely on the cathode because the anode contains no pre‐installed Li. Thus, any irreversible Li loss is directly related to progressive capacity loss [15, 16]. To address this intrinsic problem of AFASSBs, Lee et al. proposed a sacrificial cathode additive, Li_2_Cu_0.6_Ni_0.4_O_2_ (LCNO), to replenish Li during early cycling (Figure 9a) [93]. Critically, LCNO exhibits behavior that is dependent on the electrochemical stability window. Over 4.3–1.5 V, it delivers a high initial charge capacity of 393.3 mAh g^−1^ and an ICE of 84.0%, indicating reversible reactions. In contrast, within 4.3–3.0 V [101], it still delivers 393.3 mAh g^−1^ on the first charge but exhibits a very low ICE (5.6%). The pronounced irreversible capacity loss in this voltage range stems from structural changes that occur during the initial charge. Specifically, LCNO undergoes irreversible structural changes from an orthorhombic structure to a layered, electrochemically inactive cubic rock‐salt structure. Such structural changes lead to a lowering of stability and energy density compared to the pristine LCNO. Nevertheless, these electrochemical characteristics of LCNO enable substantial Li release during the initial charge, followed by minimal participation in subsequent electrochemical reactions. This behavior clearly demonstrates the sacrificial nature of LCNO. Furthermore, Park and Song et al. demonstrated the feasibility of LCNO for AFASSBs. The cell without LCNO exhibits a capacity retention of 67.0% after 50 cycles, whereas the 10% LCNO containing cell retains 82.7% of its capacity after 50 cycles, thereby clearly demonstrating the beneficial role of LCNO as a sacrificial cathode additive (Figure 9a).

In addition, Park et al. reported a coupled strategy for replenishing active Li and stabilizing interfaces [94]. The design integrates: (1) a sacrificial Li source, Li_3_N embedded in the NCM cathode composite, and (2) a Li‐free In metal layer as the anode to avoid growth of Li dendrite (Figure 9b). During the first charge of a composite electrode comprising Li_3_N, LPSCl, and a conductive agent, a voltage plateau near 3.0 V (vs. Li/Li^+^) is observed, consistent with the decomposition of Li_3_N. SEM and differential electrochemical mass spectroscopy (DEMS) analyses corroborate that Li_3_N decomposes under ASSB conditions, releasing Li into the cell. Beyond serving as a Li reservoir, increasing the Li_3_N fraction raises the internal pressure of the cell. The mechanism is that Li is released from Li_3_N alloys with the In layer, inducing volume expansion; within the confined stack, this translates into higher adequate stack pressure. The elevated pressure improves interparticle contact, enhances ionic conductivity, and increases capacity while supporting more uniform plating/stripping. Combining a Li_3_N sacrificial cathode with a Li‐free In anode mitigates Li inventory loss and enables stable cycling.

Ni‐rich layered oxides have attracted considerable attention in ASSBs owing to their high specific capacity. However, interfacial instability between Ni‐rich layered oxides and sulfide‐based SSEs during cycling induces both cathode structural degradation and SSE decomposition [102]. To address these issues, the Zhou group introduced a Li_5_FeO_4_ (LFO) coating on Ni‐rich layered oxide (NCM811), which simultaneously functions as an interfacial protective layer and a pre‐lithiation agent (Figure 9c) [95]. The LFO was synthesized via a conventional solid‐state reaction, a cost‐effective method [103]. The introduced LFO layer suppresses direct physical contact between NCM811 and the SSE, thereby mitigating the formation of insulating interfacial by‐products such as Li_2_S and P_2_S_x_ increase interfacial resistance. As a result, the LFO coating enables the formation of a stable and conductive interface. In addition, in situ pressure variation measurements were performed to evaluate the structural stability. In ASSBs, stack pressure is closely correlated with solid‐solid contact. NCM811 undergoes significant volume expansion and contraction during cycling, and excessive dimensional changes induce mechanical stress and particle cracking, as shown in Figure 9c. Notably, the introduction of LFO reduces pressure fluctuations during cycling. The Li supplied from the LFO as a pre‐lithiation agent mitigates volume changes under fixed‐gap conditions, thereby preserving the mechanical integrity of the cathode and enabling reversible lithiation and delithiation. Although Xu et al. employed indium metal as the counter electrode, LFO releases a substantial amount of Li during the initial charge and contributes minimally to reversible capacity in subsequent cycles, similar to LCNO and Li_3_N. Therefore, LFO can also possibly function as a sacrificial cathode additive in AFASSBs.

Collectively, AFASSBs have been introduced as a promising strategy to maximize both gravimetric and volumetric energy density by eliminating pre‐installed Li. However, this design inherently results in a limited Li inventory, establishing a fundamental trade‐off between energy density and cycle stability. In such systems, any irreversible Li loss directly leads to rapid capacity decay, thereby posing a critical challenge for practical implementation. The strategies introduced above demonstrate that sacrificial cathode additives provide an effective means of compensating for irreversible Li loss during early cycling. By supplying additional Li without participating in subsequent electrochemical reactions, these additives mitigate Li inventory loss while preserving the high‐energy‐density advantage of AFASSBs. Therefore, sacrificial cathode additives represent a viable strategy to balance the intrinsic trade‐off in AFASSBs, enabling both enhanced energy density and improved cycle stability.

#### 3.3.2 Modification of Solid‐State Electrolytes

To address the interfacial instability arising from the high reactivity between sulfide‐based SSEs and Li, as well as the non‐uniform Li plating/stripping behavior, modification of the SSE itself, as a key component of AFASSBs, has also been explored by several research groups [96, 97, 98].

**FIGURE 10:**
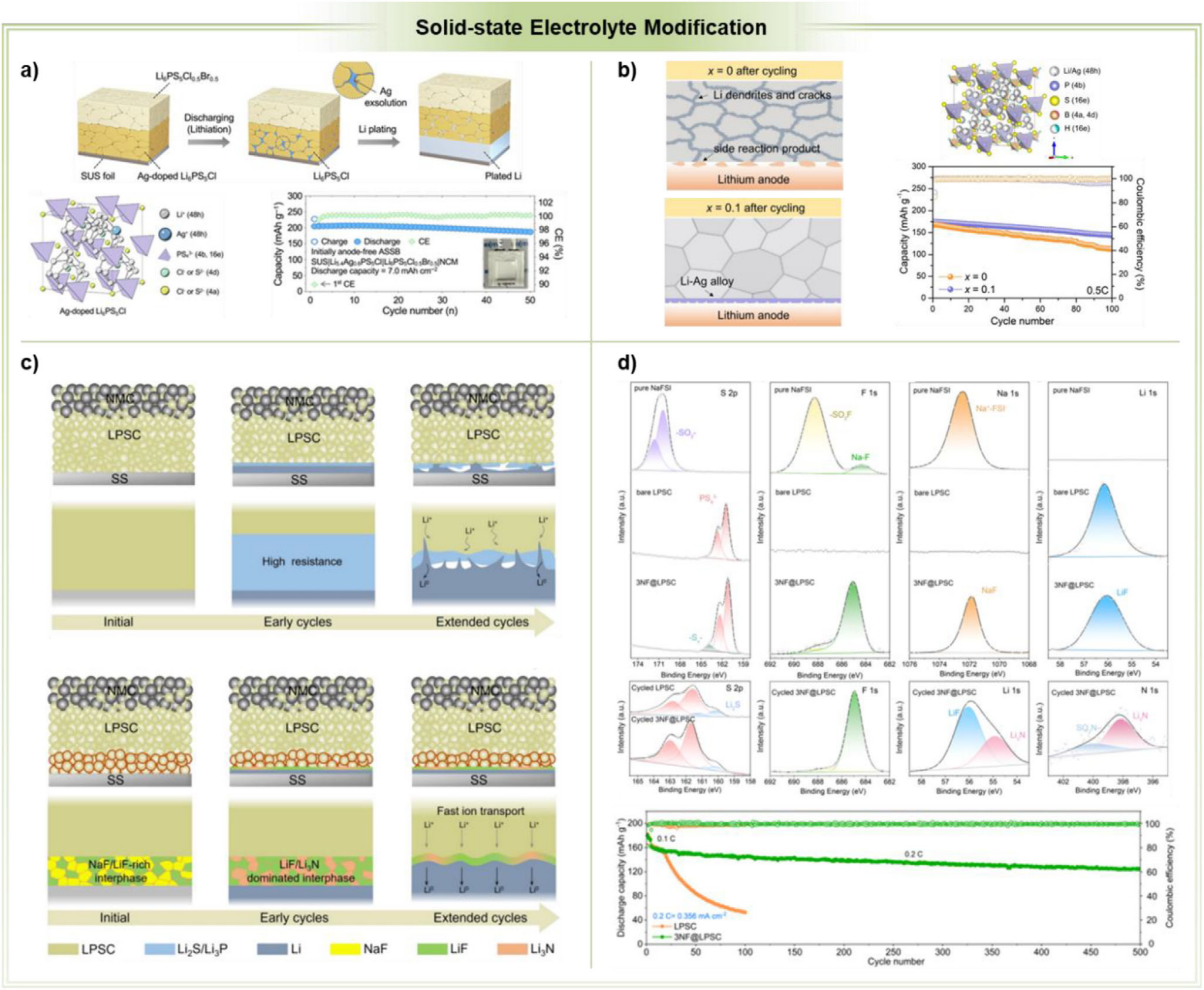
Strategies dealing with modification of SSEs for performance enhancements in AFASSBs: (a) Schematic illustration of Ag exsolution‐induced Li plating mechanism. Crystal structure of LPSCl occupied by Ag^+^ dopant ions. Cycle performance of the pouch‐type AFASSB at 60°C. Reproduced with permission [96]. Copyright 2025, Springer Nature. (b) Schematic illustration of the interfacial reactions between Li metal and the SSE of *x* = 0 and *x* = 0.1, respectively. Crystal structure of synthesized SSE and cycle performance of ASSBs composed of both SSE of *x* = 0 and *x* = 1 at 0.5 C. Reproduced with permission [97]. Copyright 2025, American Chemical Society. (c) Schematic illustration of time‐evolving hetero‐alkali interphases in AFASSBs. Reproduced with permission [98]. (d) XPS spectra of cycled LPSC and 3NF‐LPSC (top) and cycle performance of AFASSBs incorporating 3NF‐LPSCl (bottom). Reproduced with permission [98].

Choi et al. proposed an innovative route to mitigate nonuniform Li plating/stripping and dendrite formation in AFASSBs by using an Ag‐doped Li‐argyrodite as part of a bilayer SSE; an Ag‐doped argyrodite layer and an undoped argyrodite layer (Figure 10a) [96]. During charging, Ag^+^ ions in the doped layer diffuse along grain boundaries and pores and are electrochemically reduced in electron‐rich regions, triggering Ag exsolution and the in situ formation of nanometric Ag seeds. As a well‐known lithiophilic metal, Ag then alloys with Li, providing uniform nucleation sites that suppress dendrites, promote even Li deposition, and fill internal voids within the SSE, lowering interfacial resistance [61, 104]. Ag can re‐dissolve and migrate to the SSE layer upon discharge. With this bilayer SSE, pouch‐type cells delivered a high areal capacity of 7.0 mAh cm^−2^ at 0.7 mA cm^−2^ under a practical stack pressure of 2 MPa, retaining 95% capacity after 50 cycles and achieving a volumetric energy density of 1312 Wh L^−1^.

Later, Noh et al. reported an Ag‐substituted borohydride argyrodite sulfide SSE that delivers superior interfacial stability in ASSBs (Figure 10b) [97]. Focusing on the unstable interfacial reactions between Li metal and the argyrodite family Li_6_PS_5_X (X = Cl, Br, I). They proposed partial substitution of Li with Ag in a BH_4_^–^‐based argyrodite to improve compatibility with Li metal. The Ag‐substituted BH_4_^–^ argyrodite SSE, Li_6–x–γ_Ag_x_PS_5–γ_(BH_4_)_1+γ_, was successfully synthesized via a simple two‐step ball‐milling process without heat treatment. XRD combined with Rietveld refinement confirmed the incorporation of Ag into the crystal structure. Interfacial stability at the SSE‖Li interface was evaluated using Li symmetric cells. The cell employing the Ag‐substituted SSE at *x* = 0.1 exhibited stable Li plating/stripping for more than 500 h at 0.5 mA cm^−2^ and more than 300 h at 2 mA cm^−2^, reaching a CCD of 2.9 mA cm^−2^. XPS further revealed the formation of an Ag‐Li alloy at the interface during Li plating/stripping. In contrast, the Ag‐free SSE (*x* = 0) accumulated resistive byproducts such as Li_2_S and reduced phosphorus species after cycling, which increased interfacial resistance and promoted dendrite formation. Finally, ASSB with Ag‐substituted (*x* = 0.1) SSE, LiNbO_3_‐coated NCM811 cathode, and a Li‐In alloy delivered an initial discharge capacity of 204.0 mAh g^−1^ at 0.05 C and retained 82.2% of its capacity after 100 cycles at 0.5 C. Such substitution strategies are expected to enhance performance when implemented in AFASSBs as well.

In addition to the strategy of Ag substitution in SSEs, the H. Zhu group reported an alternative approach involving the introduction of sodium bis(fluorosulfonyl)imide (NaFSI) as an additive into the SSEs to achieve interfacial stabilization (Figure 10c) [98]. In AFASSBs, Li ions are transported from the cathode to the anode during each cycle, where Li is continuously plated onto the current collector. As a result, fresh Li surfaces are persistently exposed throughout cycling. Under these conditions, a static passivation layer formed during the initial stages is insufficient to effectively stabilize the newly formed Li surface. Consequently, the freshly plated Li comes into direct contact with the SSE, leading to undesirable side reactions and subsequent degradation of cycle performance. To address this issue, the authors proposed that the interphase in AFASSBs should be kinetically programmed, evolving progressively during cycling rather than remaining static. Based on this concept, they introduced a NaFSI additive to form a time‐evolving hetero‐alkali interphase that initially forms a NaF/LiF‐rich layer and subsequently evolves into a LiF/Li_3_N‐rich layer.

The underlying mechanism can be described as follows. The xNaFSI‐LPSC was synthesized via annealing. During heat treatment, NaFSI chemically interacts with nucleophilic sulfide species present on the LPSC surface, leading to cleavage of the S─F bond in the FSI^−^ anion. The released F^−^ anions are highly reactive and subsequently combine with Li^+^ in LPSC and Na^+^ derived from NaFSI to form thermodynamically stable LiF and NaF. The authors confirmed the formation of these species using XPS, as shown in Figure 10d. Prior to cycling, the Na 1s spectrum exhibits a peak shift to lower binding energy compared to pristine NaFSI, indicating the formation of NaF. In the Li 1s spectrum, the presence of LiF further demonstrates that Li^+^ participates in the formation of LiF, thereby contributing to initial interfacial stabilization. After cycling, the S 2p spectrum shows a reduced formation of Li_2_S compared to cycled pristine LPSC, indicating suppression of reductive decomposition of the SSE. In addition, the F 1s spectrum reveals an increased abundance of LiF/NaF‐rich species, while the Li 1s spectrum shows the emergence of Li_3_N peaks alongside LiF. These observations indicate the formation of N‐containing inorganic products. Collectively, these results demonstrate the formation of a time‐evolving inorganic interphase during cycling. Full‐cell evaluation in an anode‐free configuration further confirms the effectiveness of the NaFSI‐modified SSE. The cell exhibits a capacity retention of 77.6% after 500 cycles at 0.2 C, demonstrating significantly improved cycle stability.

#### 3.3.3 Cell Assembly and Cycle Conditions

While the previous sections have focused on improving performance in AFASSBs by modifying cell components (i.e., interface of current collector, pressure‐regulating buffer layers, sacrificial cathode additives, and SSE modification) to address intertwined chemo‐mechanical failure modes, this section instead highlights strategies that optimize cell assembly and cycle conditions rather than altering specific components within AFASSBs.

**FIGURE 11:**
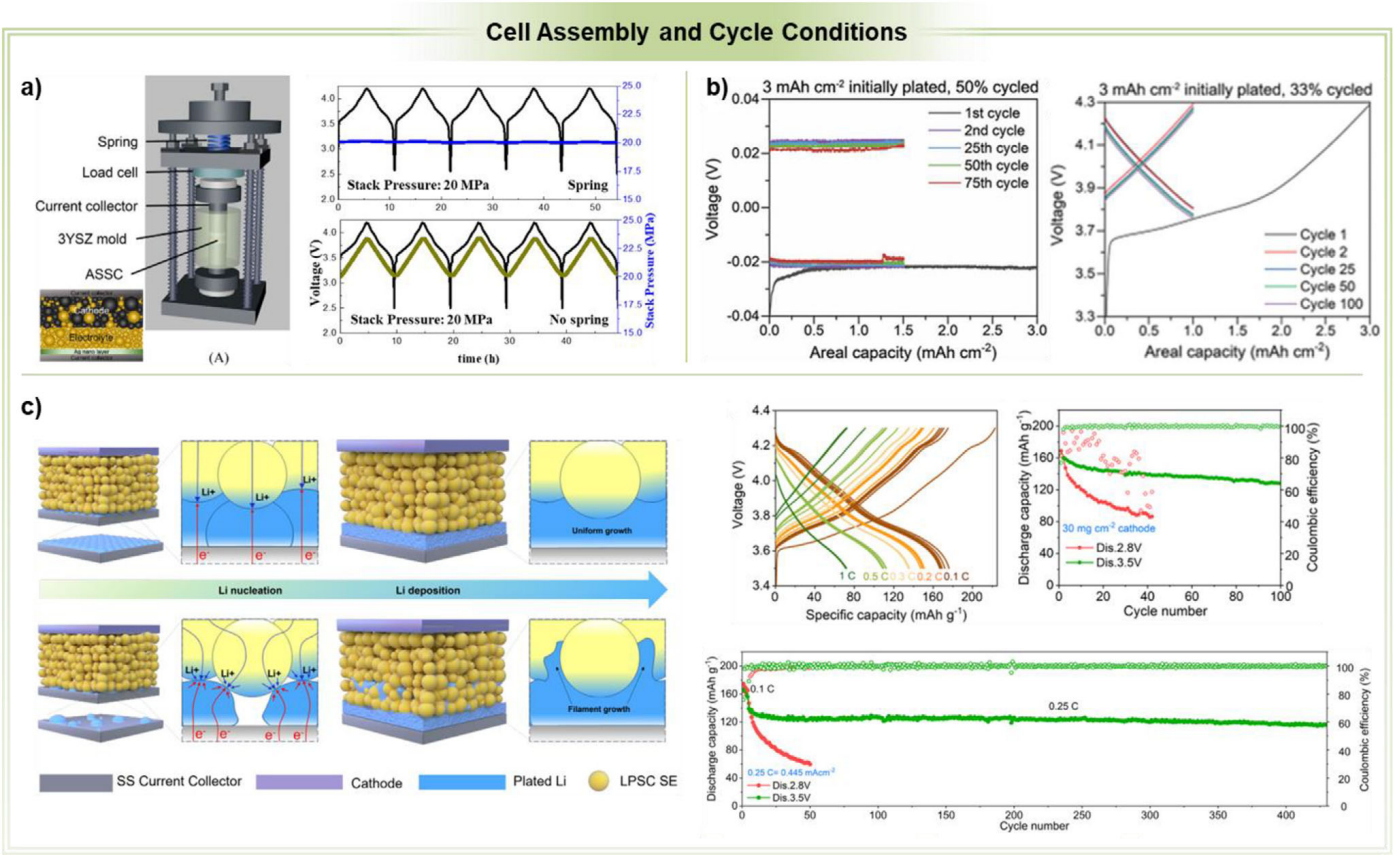
Representative strategies regarding cell assembly and cycle conditions to enhance cycle performance of AFASSBs: (a) Schematic illustration of spring‐equipped cell assembly in AFASSBs and voltage and pressure profiles for each with/without spring at stack pressure of 20 MPa. Reproduced with permission [99]. Copyright 2023, The American Ceramic Society. (b) Schematic illustrations of the mechanism of accelerated short circuiting in AFASSBs driven by local Li depletion and voltage profiles of AFASSBs cycled under capacity‐limited conditions for both Cu/LPSC/Li half‐cell and Cu/LPSC/NCM full‐cell, respectively. Reproduced with permission [49]. Copyright 2023, Wiley‐VCH GmbH. (c) Schematic illustration of discharge cutoff voltage‐controlled strategies, voltage profiles, and cycle performance of AFASSBs incorporating discharge cutoff voltage protocol. Reproduced with permission [100]. Copyright 2026, Chemrxiv.

AFASSBs, which resemble All‐solid‐state Lithium Metal Batteries (ASSLMB), undergo significant volume changes during repeated Li plating/stripping [19]. Unlike Li symmetric cells, where the net volume change is negligible, full‐cell configurations cannot compensate for the dimensional variations between the cathode and anode during cycling. As a result, the imbalance in volume change increases stack pressure. In the fixed‐gap designs, this elevated pressure further increases the likelihood of premature short‐circuiting. To address this issue, Ham et al. designed a constant‐pressure cell architecture incorporating springs to regulate pressure variations during cycling, as illustrated in Figure 5b [66]. Similarly, Park et al. proposed a spring‐equipped assembly to alleviate stress induced by volume changes and to maintain constant pressure during cycling in AFASSBs [99]. They fabricated a cell composed of an NCM811, LPSCl, and a Cu current collector coated with an Ag layer. The designed assembly incorporated a load cell to measure stack pressure and a spring that continuously compensates for volume fluctuations during cycling (Figure 11a). By incorporating a spring‐equipped assembly, the authors aimed to maintain constant pressure throughout cycling, thereby alleviating stress associated with volume changes in the electrodes. Under a low stack pressure of 10 MPa, both spring‐equipped and spring‐free cells exhibited rapid capacity degradation of approximately 88% and 96%, respectively, after 50 cycles. In contrast, at 25 MPa, the spring‐equipped AFASSB showed the best cycle stability, maintaining 97.5% of its capacity after 50 cycles. These results demonstrate that sufficient stack pressure is essential for the stable operation of AFASSBs employing Li metal anodes and sulfide‐based SSEs, and that managing the stress induced by volume changes is crucial for ensuring interfacial and electrochemical stability.

Beyond cell assembly strategies, Lewis et al. systematically investigated short‐circuit acceleration induced by Li depletion in AFASSBs [49]. The proposed mechanism is already shown in Figure 4b. To mitigate localized Li depletion, the authors proposed partially cycling the cell after initial Li deposition to intentionally maintain a Li reservoir at the interface. When 3 mAh cm^−2^ of Li was initially deposited, and only 33% of the capacity was cycled, stable cycle performance was maintained up to the 100th cycle (Figure 11b).

Following a strategy similar to that proposed by the M. T. McDowell group, Wang et al. introduced a discharge‐cutoff‐voltage strategy for AFASSBs [100]. By increasing the discharge cutoff voltage from 2.8 to 3.5 V, cycling was conducted under conditions that intentionally preserve a residual Li layer, which can serve as an in situ‐formed seed layer providing uniform nucleation sites for subsequent Li plating. (Figure 11c) Electrochemical evaluation revealed that AFASSBs operating at an elevated discharge cutoff voltage delivered a discharge capacity of 116 mAh g^−1^, with a capacity retention of 83.4% after 430 cycles at 0.25 C, and an average CE of approximately 99.9%. In contrast, cells cycled at the conventional cutoff voltage of 2.8 V exhibited rapid capacity fading, retaining only 59.7 mAh g^−1^ (40.7% capacity retention) after 50 cycles. These results indicate that complete Li stripping induces Li fragmentation and interfacial void formation, leading to contact loss and non‐uniform current distribution. By intentionally maintaining a thin residual Li layer, the authors proposed a simple yet effective protocol to improve interfacial stability and enhance the overall performance of AFASSBs.

### 3.4 Critical Comparison and Design Considerations for Performance Enhancement Strategies

Section 3 encompasses a broad range of approaches, from interfacial engineering to cell assembly and cycle protocol. To enhance the practical utility of the aforementioned strategies as a design guide for AFASSBs, a critical comparison of the approaches is summarized herein, highlighting their primary mechanisms, key advantages, and potential drawbacks.

The incorporation of interfacial engineering strategies, including lithiophilic metal seed layers, conversion‐type interlayer, and carbon‐based interlayer, primarily aims to regulate Li^+^ flux and homogenize Li plating at the SSE‖current collector interface. Lithiophilic metal seed layers effectively overcome the intrinsically high nucleation energy barrier of current collector metals, thereby reducing nucleation overpotential and enabling more uniform initial Li coverage. However, these layers do not inherently prevent interfacial side reactions at the SSE‖plated Li interface, which could compromise long‐term stability [68, 69]. In contrast, conversion‐type interlayers form ionically conductive and electronically insulating phases upon initial reaction with Li. This in situ‐formed interphase not only promotes uniform Li plating but also suppresses direct contact between the SSE and plated Li, thereby improving interfacial stability. Nevertheless, a notable limitation of this approach is the limited number of suitable metal candidates that can undergo a conversion‐type reaction [56, 57]. Carbon‐based interlayers similarly contribute to uniform Li plating and effectively shift Li deposition to the interlayer‖current collector interface [70, 71]. Extensive investigations have been conducted to elucidate the underlying mechanism governing Li deposition behavior in these systems. Although a definitive mechanism has not yet been fully elucidated, it is generally understood that Li plating behavior arises from the interplay of multiple factors. These include the properties of alloying‐forming metals (e.g., grain size and lithiophilicity), current density, temperature, structural characteristics of carbon materials (e.g., 2D Li diffusion along stacked graphene basal planes in graphite vs. the highly disordered nature of amorphous carbon), and interfacial adhesion at each interface.

The implementation of pressure‐regulating buffer layers has been employed as an effective strategy to address critical challenges in AFASSBs, including the formation of electrochemically inactive regions, current constriction, Li filament growth, and dead Li, which originate from contact loss between the SSE and residual Li on the current collector during stripping. By incorporating various components such as carbon felt, MXene/Mg, and three‐dimensional Li hosts, these approaches can accommodate volume changes during cycling and maintain intimate interfacial contact at the SSE‖current collector interface [86, 87, 88, 89]. As a result, interfacial stability is improved, leading to enhanced cycle performance. However, these strategies inherently involve a trade‐off with one of the key advantages of AFASSBs, namely the maximization of energy density, and a similar limitation is also observed in strategies that intentionally retain residual Li, as discussed in Section 3.3.3. Given that Li plating predominantly occurs at the SSE‖current collector interface without additional interlayers, freshly plated Li inevitably comes into direct contact with SSE, causing thermodynamically driven reductive decomposition. Instead of incorporating interlayers to address this issue, strategies for modifying SSEs have also been explored. A comparative summary of the performances of the various strategies discussed throughout this review is provided in Table 1.

**TABLE 1:** Comparative summary of representative strategies in AFASSBs, summarizing key performance metrics including an areal capacity, stack pressure, operating temperature, and retention.

Performance enhancing strategies	Anode‐free architecture	Cell type	Areal capacity [mAh cm^−2^]	Stack pressure [MPa]	Operation temperature [°C]	Retention	Refs.
Interlayer engineering	In doped Ag@SUS	Pellet cell	1.45	∼30	50	80.2% after 250 cycles at 1 C	[68]
Interlayer engineering	Ag‐ZnO@SUS	Pellet cell	3	20	RT	80.8% after 100 cycles at 0.2 C	[69]
Interlayer engineering	AgF@SUS	Pellet cell	7	20	RT	85.4% after 50 cycles at 0.1 C	[57]
Interlayer engineering	Cu_2_Te@Cu	Pellet cell	0.96 – 1.92	∼13	RT	80.0% after 50 cycles at 0.2 C	[56]
Interlayer engineering	Ag‐C@SUS	Pouch cell	6.8	2 – 4	60	89.0% after 1000 cycles at 0.5 C	[70]
Interlayer engineering	Ag‐CB@SUS	Pouch cell	4.2	∼ 4	60	86% after 700 cycles at 0.5 C	[71]
Pressure‐regulating buffer layers	Carbon Felt	Pellet cell	3	7.5	RT	55.4% after 100 cycles at 0.2 C	[86]
Pressure‐regulating buffer layers	MXene/Mg	Pellet cell	2.8	2	RT	57.7% after 30 cycles at 0.1 C	[87]
Pressure‐regulating buffer layers	TiN NTs	Coin cell	3.2	∼0.6	60	78.3% after 600 cycles at 0.3 C	[89]
Sacrificial cathode	LCNO	Pellet cell	4	N/A	N/A	82.7% after 50 cycles at 0.1 C	[93]
Sacrificial cathode	Li_3_N	Pellet cell	4	N/A	55	75.8% after 200 cycles at 0.2 C	[94]
Sacrificial Cathode	LFO	Pellet cell	3.7	N/A	N/A	81% after 80 cycles at 0.2 C	[95]
SSE modification	Ag‐doped Li‐argyrodite SSE	Pouch cell	7.0	2	60	95.0% after 50 cycles at 0.1 C	[96]
SSE modification	Ag‐substituted borohydride argyrodite SSE	Pellet cell	2.3	∼50	30	82.2% after 100 cycles at 0.5 C	[97]
SSE modification	NaFSI	Pellet cell	1.78	∼15	RT	77.6% after 500 cycles at 0.2 C	[98]
Additional equipment	Spring‐equipped cell	Pellet cell	2.08	25	80	97.3% after 50 cycles at 0.2 C	[99]
Cycle Condition Protocol	Ag@SUS	Pellet cell	1.78	15	RT	83.4% after 430 cycles at 0.25 C	[100]

Overall, although each strategy contributes to performance enhancement through distinct mechanisms, no single approach fully resolves the multifaceted challenges associated with AFASSBs. Therefore, a synergistic integration of multiple strategies, combining controlled Li deposition, interfacial stabilization, and suppressing void formation, is essential for achieving stable and high‐performance AFASSBs.

## 4 Conclusion

This work thoroughly reviewed four critical interfacial instability factors that hinder the commercialization of sulfide‐based ASSBs. First, regarding chemical stability, atmospheric exposure of SSEs leads to the evolution of toxic H_2_S gas, which can cause catastrophic corrosion of the Cu current collectors. Potential chemical interactions between the solid electrolyte and slurry‐processing components, such as solvents and binders, must also be carefully evaluated, as they can substantially affect the interfacial stability of the system. Second, thermal stability analysis revealed that the intrinsic thermal vulnerability of specific SSE compositions, alongside the poor stability of the interface layer formed during Li plating, poses a significant threat to ASSB safety. Third, from an electrochemical perspective, functional defects within the SEI formed in the narrow stability window, particularly non‐uniform electronic/ionic conductivity, are the core cause of current crowding, ultimately leading to catastrophic Li dendrite growth and cell short‐circuiting. Finally, mechanical instability, stemming from inadequate control over pressure non‐uniformity and volume changes during Li plating/stripping, accelerates the formation of dead Li and severely degrades long‐term performance.

Ultimately, achieving the high‐energy‐density potential of AFASSBs requires an integrated approach that simultaneously addresses these multifaceted instabilities. Current research efforts are primarily concentrated on the following three key strategies:

**1 Advanced interfacial layer technology**: To concurrently overcome electrochemical instability and mechanical fragility, highly engineered interfacial layers should be introduced between the SSE and the current collector. These layers must maintain high Li‐ion conductivity while suppressing electronic conductivity to block parasitic reactions. However, achieving these two functions simultaneously remains a major challenge because interfacial materials that promote ion transport do not always provide sufficient electronic insulation or long‐term chemical stability. In addition, the interfacial layer should remain chemically and structurally stable during repeated Li plating/stripping. From a mechanical perspective, developing robust buffer layers with sufficient elasticity and strength is also crucial for mitigating local stress concentrations and accommodating interfacial deformation. Such mechanical regulation plays an important role in ensuring uniform Li nucleation and growth. Therefore, the rational design of multifunctional interlayers that can simultaneously regulate transport behavior, interfacial reactions, and stress evolution will be an important direction for future research. These layers are ultimately expected to alleviate interfacial heterogeneity and prevent dendrite formation by suppressing localized current concentration.

**2 Development of highly stable SSE compositions and processes**: Improving the intrinsic stability of sulfide SSE compositions is imperative to suppress H_2_S evolution and maximize thermal stability. Research should be expanded to compositions containing the relatively stable PS_4_^3–^ ion rather than the P_2_S_7_^4–^ ion. Furthermore, for an intrinsically unstable SSE such as Li_7_P_3_S_11_, the commercial viability must be explored by minimizing atmospheric exposure and strictly controlling dry‐processing conditions. Optimizing cell manufacturing processes in inert‐gas environments and introducing novel binding techniques are required to prevent SSE decomposition and direct reactions with the Cu current collector.

**3 Active pressure management and cell design**: A robust system capable of predicting and actively responding to volume changes during cell operation is essential to minimize the inevitable generation of dead Li in anode‐free systems. This involves incorporating flexible jigs based on springs or fluid dynamics into the cell structure to ensure uniform stack pressure across large‐area cells and accommodate the volumetric expansion/contraction during Li plating and stripping. Ultimately, constructing an integrated cell architecture in which the SSE, current collector, and pressure management system operate synergistically will be the final crucial step toward commercializing safe, long‐lasting AFASSBs.

From a manufacturing perspective, however, the commercialization of sulfide‐based AFASSBs remains highly challenging, particularly because scaling such systems from laboratory‐scale demonstrations to pilot‐scale production is far from straightforward. Nevertheless, sulfide‐based AFASSBs may still offer several practical advantages in manufacturability. One representative process‐level advantage of solid‐state batteries is the feasibility of bipolar stacking, which can improve the overall packing density and module‐level energy density compared with the bi‐layer architecture generally adopted in LIBs [105]. In addition, adopting an anode‐free design may provide further processing benefits. Cheng et al. demonstrated that in anode‐free thin‐film solid‐state batteries manufactured via scalable roll‐to‐roll processes, Li metal forms in situ on the stainless‐steel current collector during initial charging, eliminating the need for a separate anode deposition step and reducing the risks associated with handling highly reactive Li metal during fabrication. Because no pre‐existing Li metal is present during processing, concerns about Li melting or degradation at elevated temperatures are also alleviated, enabling compatibility with high‐temperature post‐processing, such as reflow soldering on printed circuit boards at temperatures up to 235°C [106]. Although demonstrated in thin‐film batteries, these process‐level advantages may also provide meaningful implications for the pilot‐scale fabrication of sulfide‐based anode‐free cells.

At the same time, recent studies suggest that highly reactive sulfide SSEs may still be incorporated into scalable fabrication routes under carefully controlled conditions. Singer et al. showed that sulfide solid electrolytes can be applied to wet‐coating slurry processes and fabricated into thin‐film‐level separators, although the rheological behavior strongly depends on particle size and associated hydrodynamic properties [107]. These results imply that sulfide SSEs may be compatible with industrial roll‐to‐roll manufacturing when chemically appropriate solvents are employed. However, the same group also reported particle cohesion failure at temperatures above 80°C, along with decreased adhesion strength at higher drying temperatures and drying rates [108]. Such findings suggest that, compared with conventional LIB manufacturing, sulfide‐based ASSBs may require lower‐temperature, slower‐drying conditions, highlighting the need to re‐optimize existing slurry‐casting routes or develop entirely new manufacturing strategies. In parallel, dry‐electrode processing and advanced printing methods have also emerged as promising alternatives. Tan et al. suggested that minimizing the binder content in SSE film fabrication can suppress the increase in Li^+^ diffusion‐path tortuosity caused by film‐like binder precipitation during wet dispersion [29, 105]. Likewise, Doerrer et al. demonstrated layer‐by‐layer spray printing of LPSCl electrolyte layers, catholyte layers, and Ag–C interlayers for anode‐free solid‐state battery architectures, while maintaining high ionic conductivity and reducing electrolyte thickness to as low as 10 µm [109]. Such approaches may mitigate some of the limitations of conventional slurry casting by improving microstructural control, coating uniformity, and large‐area processability.

Despite these advances, ensuring uniformity and stable performance at scales beyond pouch cells remains a major challenge for sulfide‐based AFASSBs. As discussed in the pressure‐related failure mechanisms above, fabrication pressure is directly linked to cell performance, yet most previous studies have focused on small‐area, pellet‐type test cells. Among the pressing routes discussed by Tan et al., continuous line pressing inherently introduces non‐uniformity, whereas uniaxial area pressing and isostatic pressing are costly and difficult to implement in pouch‐level cell fabrication [105]. In the same study, however, ASSBs employing a module‐housing structure based on springs and gaskets achieved a more uniform stack‐pressure distribution, and a 10 kWh system exhibited volumetric efficiency that surpassed that of LIBs at pressures below 5 MPa. Overall, these results suggest that sulfide‐based ASSBs may offer greater advantages in volumetric efficiency than in gravimetric efficiency at large scale, indicating strong potential particularly for stationary storage applications.

In summary, while AFASSBs represent the most transformative path toward achieving high‐energy density, the inherent chemical, electrochemical, mechanical, and manufacturing‐related fragilities of sulfide SSEs continue to impede their commercialization. Therefore, a multidimensional research strategy that integrates materials, interfacial engineering, cell design, and scalable processing is required, with priority given to overall interfacial stability rather than the isolated optimization of individual components. This must involve employing advanced interfacial coatings to restore SEI functionality, utilizing active pressure control systems to manage volume dynamics, and concurrently exploring new SSE compositions with enhanced atmospheric and thermal resilience, and developing manufacturing strategies compatible with large‐area and pilot‐scale fabrication. Only through such concerted and integrated efforts can AFASSBs secure the safety, long cycle life, and high energy density required to lead the next‐generation battery market.

## Conflicts of Interest

The authors declare no conflicts of interest.

## Data Availability

The data that support the findings of this study are available from the corresponding author upon reasonable request.
